# JAK-inhibitor treatment induces rapid yet incomplete immune reconstitution in STAT1 gain-of-function

**DOI:** 10.70962/jhi.20260009

**Published:** 2026-10-09

**Authors:** Cecilia Iglesias-Herrero, Willem Roosens, Georgina Galicia, Jonathan Cremer, Pedro Elias Marques, Timothy Devos, Leen Moens, Isabelle Meyts, Rik Gijsbers, Rik Schrijvers

**Affiliations:** 1 Advanced Disease Modelling, Targeted Drug Discovery and Gene Therapy (ADVANTAGE), Department of Pharmacological and Pharmaceutical Sciences, Faculty of Medicine, KU Leuven, Leuven, Belgium; 2 Department of Microbiology, Immunology and Transplantation, Allergy and Clinical Immunology Research Group, KU Leuven, Leuven, Belgium; 3 Department of General Internal Medicine, Division of Allergy and Clinical Immunology, University Hospitals Leuven, Leuven, Belgium; 4 Laboratory of Molecular Immunology, Department of Microbiology, Immunology and Transplantation, Rega Institute for Medical Research, KU Leuven, Leuven, Belgium; 5 Department of Hematology, University Hospitals Leuven and Laboratory of Experimental Transplantation, Department of Microbiology and Immunology, KU Leuven, Leuven, Belgium; 6 Department of Pediatrics, University Hospitals Leuven, Leuven, Belgium; 7 Laboratory for Inborn Errors of Immunity, Department of Microbiology, Immunology, and Transplantation, KU Leuven, Leuven, Belgium; 8 Leuven Viral Vector Core, Group Biomedical Sciences, Faculty of Medicine, KU Leuven, Leuven, Belgium

## Abstract

Autosomal dominant STAT1 gain-of-function (GOF) is an inborn error of immunity characterized by chronic mucocutaneous candidiasis and variable immune dysregulation. JAK inhibitors are a promising therapy, but their efficacy in STAT1 GOF remains variable. We longitudinally evaluated the clinical and molecular impact of JAK inhibitors in two STAT1 GOF patients, harboring p.R321S and p.T385M mutations, using Cellular Indexing of Transcriptomes and Epitopes by sequencing, flow cytometry, cytokine measurement, and allele-specific expression analysis in peripheral blood mononuclear cells and serum. Rapid yet incomplete immune reconstitution was observed as early as 1 week after treatment initiation. T cells rebalanced to a more homeostatic status and TCR repertoire diversity increased rapidly. However, some immune defects remained, including natural killer deficiency in one patient. The pronounced transcriptional dysregulation in monocytes was not explained by an unbalanced mutant STAT1 expression but improved with JAK-inhibitor treatment. JAK inhibitors provided significant clinical and immunological benefits to STAT1 GOF patients but did not fully restore immune homeostasis. These findings highlight the therapeutic potential and limitations of JAK inhibitors in STAT1 GOF.

## Teaser

Iglesias-Herrero et al. longitudinally assess JAK-inhibitor treatment response in two patients with STAT1 gain-of-function and show that it induces rapid but incomplete immune reconstitution, with increased TCR diversity and reversal of monocyte hyperinflammation, highlighting both therapeutic benefit and persistent immune dysregulation in this inborn error of immunity.

## Introduction

Autosomal dominant (AD) signal transducer and activator of transcription 1 (STAT1) gain-of-function (GOF) is an inborn error of immunity (IEI). Over 660 patients with this condition have been reported globally (1, 2), and >125 pathogenic variants have been identified. STAT1 GOF leads to a diverse range of infections and immune dysregulation. The most prevalent of these is chronic mucocutaneous candidiasis (CMC), which occurs in 98% of patients (1, 3, 4). However, the clinical spectrum varies and can include recurrent bacterial and viral infections, isolated lymphoproliferation, autoimmunity, vascular aneurysms, and malignancies. The last three represent the major determinants of poor prognosis (1, 5).

Initial studies in STAT1 GOF have focused on T cell–mediated immune dysregulation (3, 6, 7, 8). However, the immunological phenotype is highly variable among patients. Patients present with reduced numbers of T cells that show signs of functional exhaustion and apoptosis (9). CD4^+^ and CD8^+^ T cells exhibit an effector-skewed phenotype along with impaired Th17 differentiation, which is associated with increased susceptibility to Candida infection (10). Hypogammaglobulinemia (11, 12, 13) and autoantibody production reflect B cell dysfunction and are reported in a subset of patients. STAT1 GOF patients have also been described with impaired maturation and cytolytic and degranulation deficiencies in natural killer (NK) cells (14). Furthermore, chronic hyperinflammation in STAT1 GOF has been attributed to sustained STAT1 activation and hyperactive IFN signaling across multiple immune subsets (13). Recent studies suggest that monocytes and dendritic cells may actively drive hyperinflammation rather than simply act as bystanders in this chronic inflammatory process (5, 15). Nevertheless, the role of monocyte and macrophage populations and their interactions with other immune cells in STAT1 GOF remains poorly understood.

Conventional treatment strategies for patients with STAT1 GOF disease include chronic antifungal therapy, antibiotics for bacterial infections, and immunosuppressive therapy for autoimmunity, in addition to immunoglobulin substitution. However, recent case reports and series indicate that the reduction of STAT1 activation with JAK inhibitors can result in a significant improvement in symptoms (16, 17, 18). Hematopoietic stem cell transplantation is currently the only curative option for STAT1 GOF but has historically been associated with substantial transplant-related morbidity, graft failure, and mortality (19), although recent studies report improved overall and event-free survival, particularly when JAK inhibitors are used as bridging therapy before hematopoietic stem cell transplantation (20, 21). JAK inhibitors are also increasingly used as long-term targeted therapy. A recent multicenter retrospective study revealed that 87% of 45 patients treated with JAK inhibitor exhibited a complete or partial therapeutic benefit for typical STAT1 GOF manifestations (22). This retrospective ESID/EBMT-IEWP analysis further showed substantial heterogeneity in clinical treatment indications for JAK-inhibitor initiation, as well as in the extent and duration of the treatment responses across STAT1-GOF manifestations. This variability was compounded by markedly inconsistent dosing practices and monitoring strategies among centers and by a high frequency of adverse events (26 out of 45 patients) that in turn contributed to therapy modification or discontinuation in 14 out of 45 patients (22). Collectively, these observations underscore that not all JAK inhibitors confer uniform therapeutic benefit to all STAT1 GOF–associated disease features, thereby highlighting the urgent need for consensus-based recommendations to standardize JAK-inhibitor selection and posology, harmonize monitoring, and guide the management of treatment-related side effects.

Our research group, along with others, has shown that different STAT1 mutations can drive a GOF phenotype through distinct molecular pathways *in vitro* (23, 24). Although phenotypic diversity extends beyond genotype due to incomplete penetrance (25), recent evidence suggests that specific mutations confer more severe outcomes (26). We therefore hypothesized that mutation-specific diversity may partially underlie the heterogeneous responses to JAK-inhibitor treatment (23, 24, 27). Indeed, previous studies showed that JAK-inhibitor treatment can reverse some, but not all, of the immunological and molecular abnormalities caused by STAT1 GOF (16, 17, 18, 28). Whereas the majority of cells from STAT1 GOF patients exhibit elevated levels of phosphorylated STAT1 (pSTAT1) upon IFN type I and type II stimulation *in vitro*, JAK inhibitors can reduce STAT1 phosphorylation and normalize IFN-stimulated gene (ISG) expression, probed by qPCR of 6–20 ISGs in a cohort of 14 patients (29). In T cells from STAT1 GOF patients evaluated *ex vivo*, JAK inhibitors reduce T cell apoptosis (9), enhance cell survival in lymphopenic patients, correct Th1 polarization, and expand Th17 subsets, concomitant with clinical amelioration of CMC (30). Inflammatory biomarkers, such as CXCL10, also decline upon treatment (31). However, not all STAT1 GOF features responded equally. For instance, NK cell maturation and cytotoxicity only show partial improvement (32), while neutrophils maintain their pro-inflammatory cellular program despite JAK-inhibitor treatment (33). It remains uncertain whether mutation-specific mechanisms underlying the STAT1 GOF phenotype (23, 24, 27), allele-specific expression in different immune cell subsets (25), or both contribute to the complexity.

We aimed to delineate in-depth the impact and breadth of JAK-inhibitor treatment in STAT1 GOF, based on the analysis of two patients with variable phenotypes. Advances in single-cell technology have proven valuable in revealing STAT1 GOF heterogeneity. However, previous studies limited their analysis to either a single patient or mass-spectrometry–based single-cell technology (CyTOF) or single-cell RNA sequencing (scRNA-seq) excluding the treatment response evaluation (17, 34, 35). In this study, we performed multi-omics, flow cytometry, serum cytokine, and TCR repertoire analyses to compare treatment-naïve patients longitudinally in both the early and late states after the initiation of JAK inhibitor in two STAT1 GOF patients. These findings, integrated with clinical data, provide a comprehensive view of JAK-inhibitor impact on cellular and transcriptional dynamics and provide insights into both therapeutic benefit and residual immune dysregulation in STAT1 GOF.

## Results

### JAK inhibitors lead to partial clinical improvement in two STAT1 GOF patients

**Figure 1.**
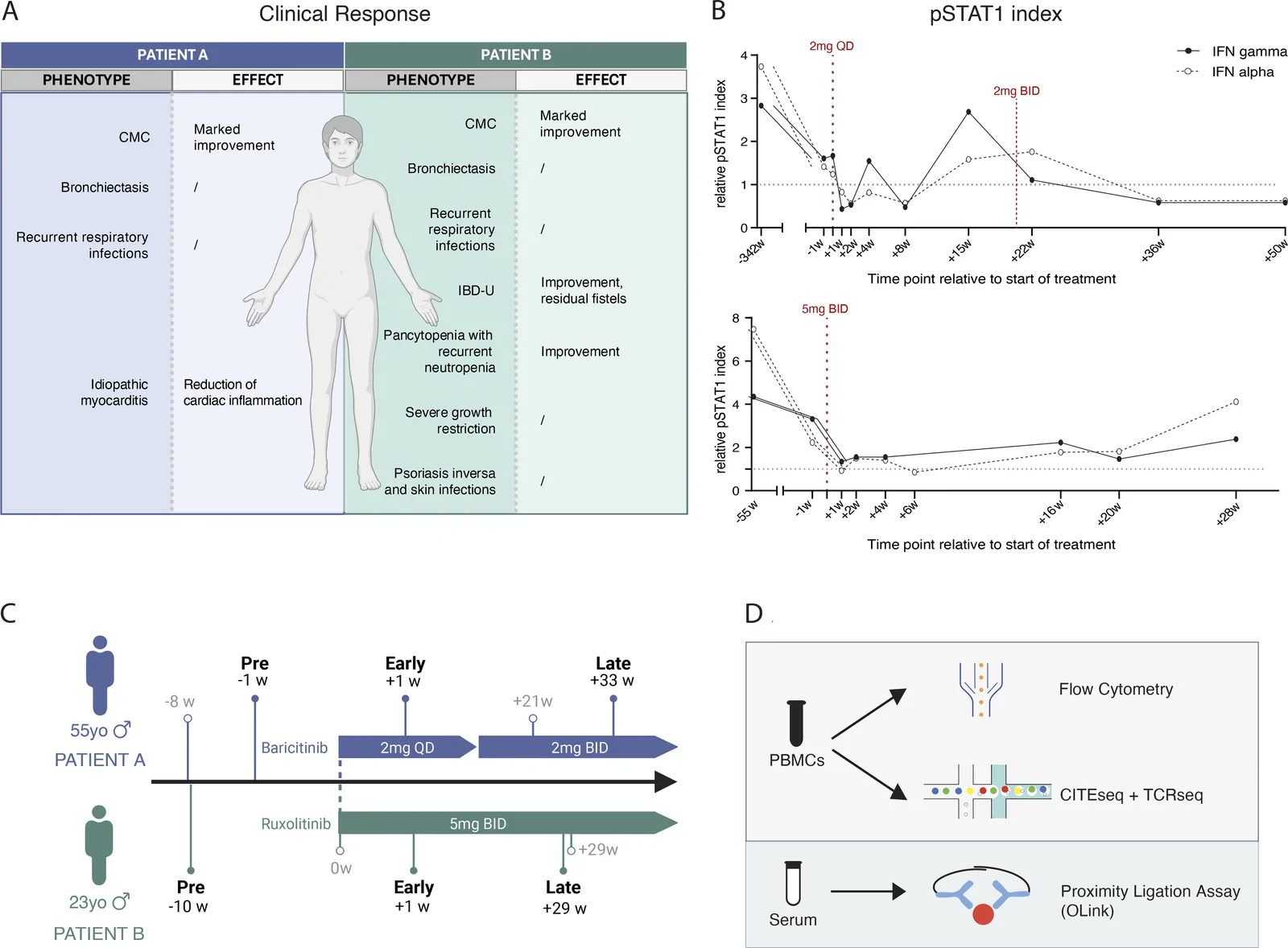
**Clinical data, STAT1 phosphorylation dynamics, and longitudinal analysis in two patients with STAT1 GOF. (A)** Patient-specific disease manifestations and corresponding response to JAK inhibitors. **(B)** Longitudinal STAT1 phosphorylation (pSTAT1) index in peripheral blood monocytes following IFN-γ and IFN-α treatment shown as relative to HC; dashed vertical red line indicates start of JAK-inhibitor treatment; dashed horizontal gray line indicates healthy reference, and x axis shows week of measurement relative to start of the JAK-inhibitor treatment. **(C)** Study schema showing visit windows: Pre (before therapy), early (∼1 wk after start), and late (∼6 mo after start). Full dots represent PBMC sampling, and empty dots represent serum sampling. **(D)** Experimental workflow. yo, years old; QD, *quaque die* = once daily; BID, *bis in die* = twice daily; TCR seq, TCR sequencing.

**Figure S1.**
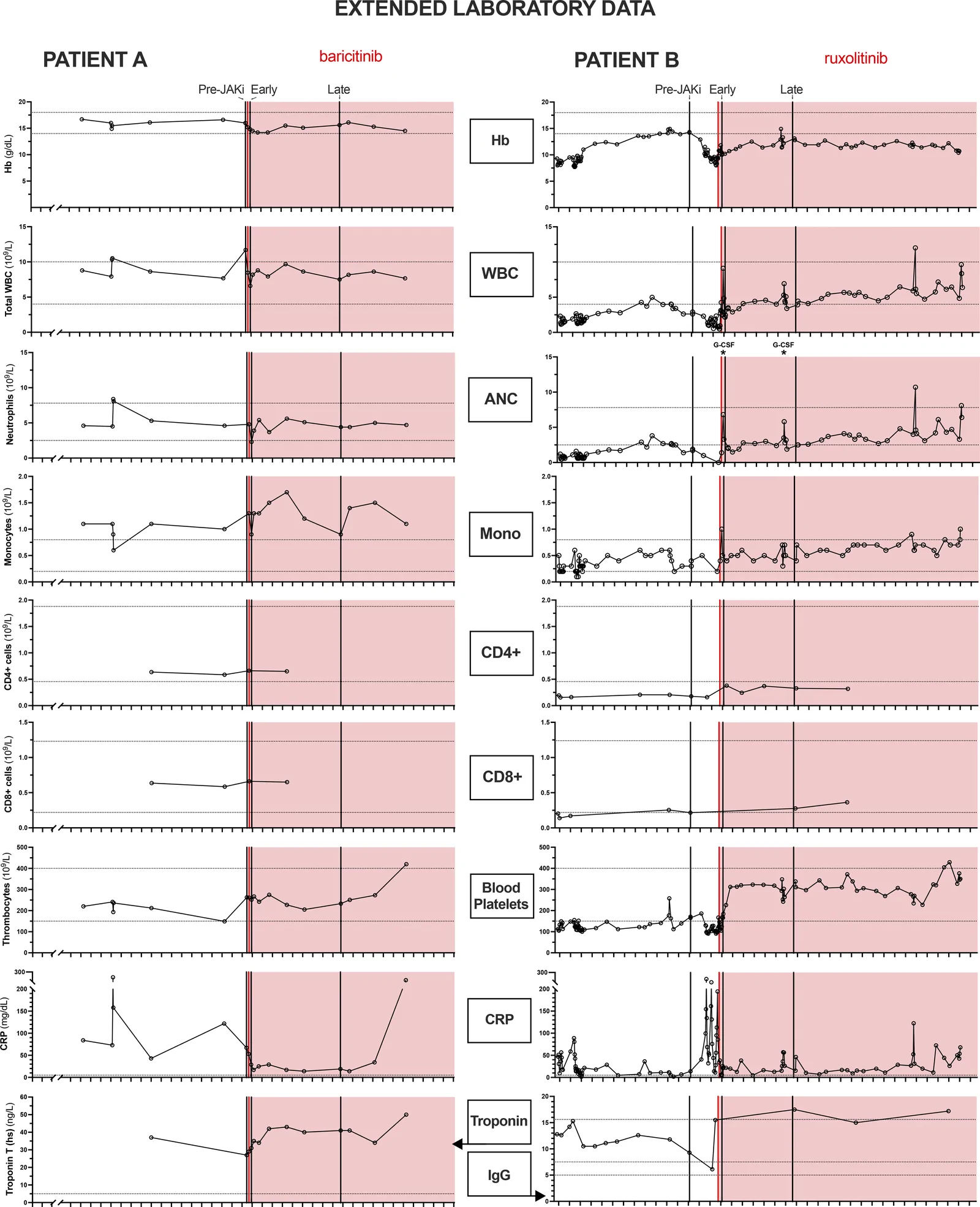
**Extended laboratory data and hematologic parameters.** Longitudinal measurements of hematologic and inflammatory parameters in two patients with STAT1 GOF mutations treated with JAK-inhibitors (baricitinib and ruxolitinib, respectively). Left panels show Patient A (baricitinib treatment) and right panels show Patient B (ruxolitinib treatment). Measured parameters include hemoglobin (Hb, g/dl), total white blood cell count (WBC, 10^9^/L), absolute neutrophil count (ANC, 10^9^/L), monocyte count (10^9^/L), CD4^+^ T cell count (10^9^/L), CD8^+^ T cell count (10^9^/L), thrombocyte count (10^9^/L), C-reactive protein (CRP, mg/dl), high-sensitivity cardiac troponin T (Troponin T (hs), ng/l), and immunoglobulin G (IgG). Asterisks (*) indicate time points where G-CSF support was administered. Individual data points are plotted with connecting lines to visualize temporal changes across the treatment course. Sampling time points are marked by a vertical black line: Pre-JAKi (baseline), early (1 wk treatment), and late (>6 mo treatment phase). Red vertical line indicates start of treatment and red shading sampling during treatment.

Patient A (c.963A>T, p.R321S) was a previously described 55-year-old male, suffering from CMC complicated by esophageal stenosis, recurrent respiratory infections, and bronchiectasis (36). Baricitinib was initiated after an incidental finding of slowly progressive idiopathic myocarditis, suspected to be an immune-mediated complication of STAT1 GOF. Infectious and autoimmune workup was unrevealing (including negative SARS-CoV-2, influenza A/B, and a broad autoantibody panel; Table S3), and no biopsy was performed. JAK-inhibitor treatment resolved CMC, reduced the frequency of respiratory infections, improved left ventricular ejection fraction, and mitigated cardiac inflammation on magnetic resonance imaging. Ultimately, the patient succumbed to fatal arrhythmia most likely due to irreversible cardiac fibrosis (Fig. 1 A). Patient B (c.1154C>T, p. T385M) is a 23-year-old male presenting with CMC, recurrent respiratory and skin infections, as well as severe immune dysregulation, including unclassified inflammatory bowel disease (IBD-U) and recurrent episodes of presumed immune-mediated pancytopenia with severe neutropenia. His phenotype was further marked by growth restriction, malnutrition, and mineral bone disease. Initiation of ruxolitinib at the age of 21 improved CMC but only led to partial stabilization of blood cell counts and limited improvement of IBD-U with residual active fistulation (Fig. 1 A). Extended patient descriptions and laboratory values are summarized in Table S1 and Fig. S1. In both patients, the longitudinal evaluation of pSTAT1 in CD14^+^ monocytes showed a noticeable reduction in STAT1 phosphorylation upon IFN stimulation (Fig. 1 B).

Neither patient had clinical evidence of active infection at sampling. CMV PCR was negative in both patients, and EBV PCR showed only very low-level viremia. Patient A additionally had negative PCR testing for HHV6, HHV8, and parvovirus B19 during the myocarditis workup. BK and JC virus were not tested because there were no suggestive clinical features.

### JAK-inhibitors mediate rapid yet incomplete reconstitution of peripheral blood mononuclear cell subsets

**Figure S2.**
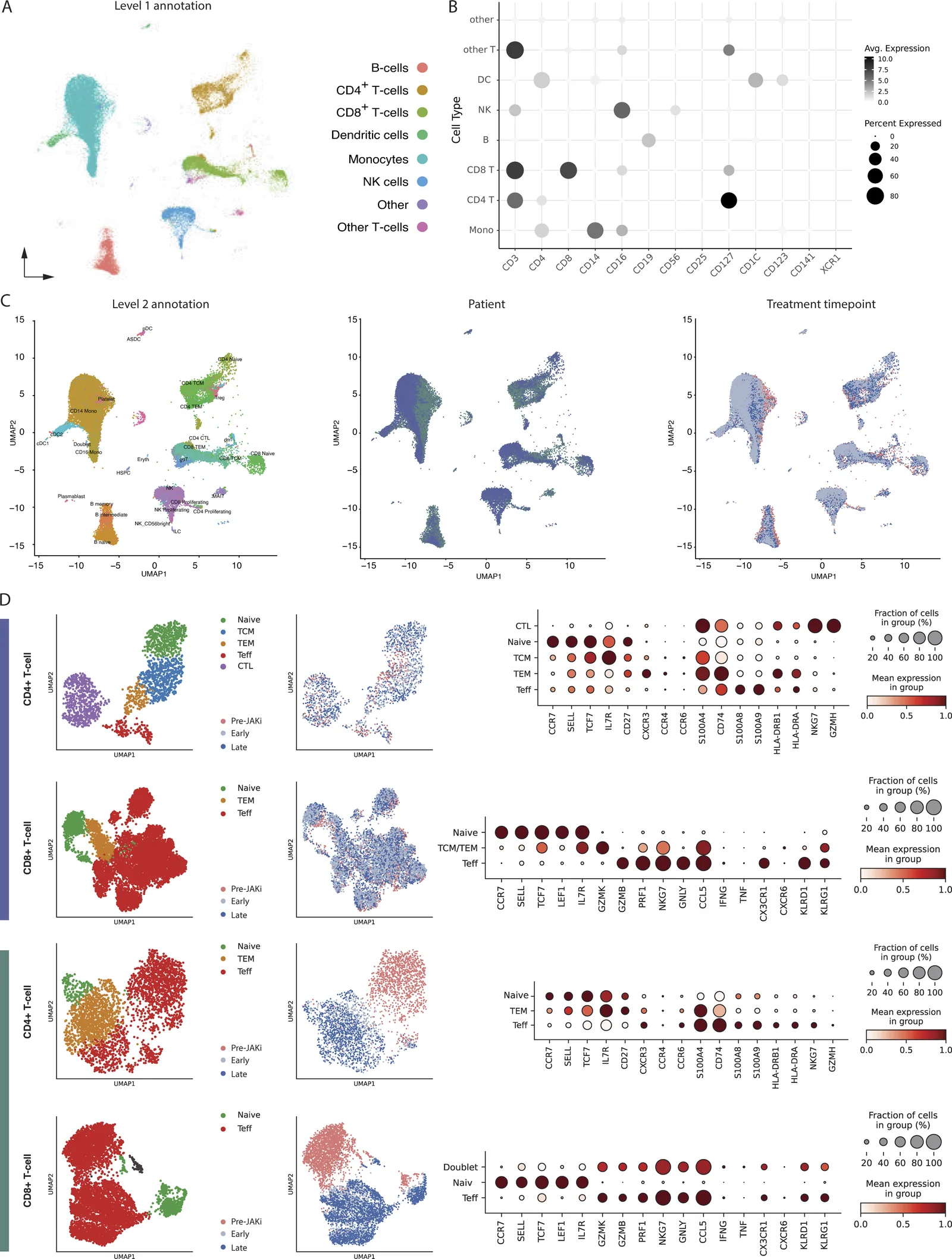
**Single-cell PBMC and T cell subset annotation. (A)** UMAP visualization showing level 1 cellular annotation of PBMCs with color-coded cell type assignments. **(B)** Dot plot displaying average expression and percent expression of selected marker genes across major immune cell types, with color intensity representing mean expression level and dot size indicating percentage of cells expressing each marker. **(C)** UMAP visualizations showing level 2 annotation (left), alongside the same UMAP space colored by patient identity (center) and treatment time point (right). **(D)** Four panels displaying CD4^+^ T cell (naïve, TCM, TEM, and Teff), CD8^+^ T cell, CD4^+^ T cell (subset analysis), and CD8^+^ T cell sub-clustering by treatment time point (left UMAP plots colored by cell phenotype and treatment phase), accompanied by corresponding dot plots (right panels) showing expression of canonical subset markers with color indicating mean expression intensity and bubble size representing the fraction of cells in each group expressing each gene. Dot plot legends display size scale (20%, 40%, 60%, 80%, and 100% of cells) and color scale (0.0–1.0 mean expression).

**Figure 2.**
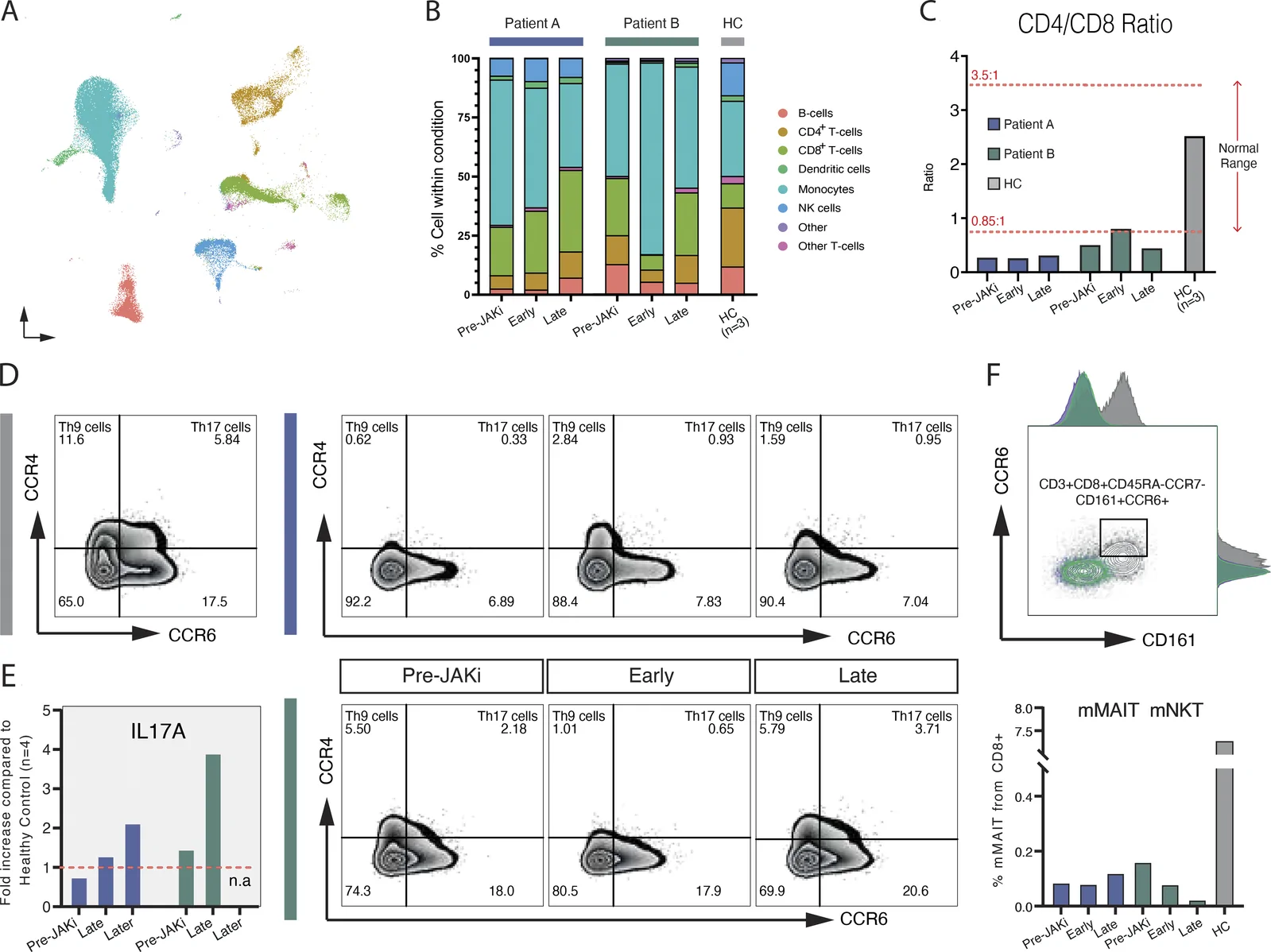
**Evolution of PBMC composition during JAK inhibition and IL17-producing cells. (A)** UMAP of all PBMCs colored by lineage annotation for visualization of major populations. **(B)** Stacked bar plot of PBMC composition showing relative abundance of PBMC lineages per sample across pre-JAKi, early, and late visits for each patient compared with a healthy reference (HC; *n* = 3). **(C)** CD4^+^/CD8^+^ T cell ratio per time point; dashed red lines marks the reference values. **(D)** Flow cytometry contour plots gating CCR6 and CXCR3 on memory CD4^+^ T cells to define Th17-like (CCR6^+^CXCR3^−^) and Th1-like (CCR6^−^CXCR3^+^) states across time points. **(E)** IL-17A production fold-increase via Olink assay, with dashed line indicating control mean. **(F)** Gating strategy for innate-like T cells using CD161 and CCR6 expression within CD3^+^CD8^+^CD45RA^−^CCR7^−^; bar plot illustrates the invariant MAIT/mNKT percentage, with a dashed red line marking the minimum healthy range observed in literature. JAKi, JAK inhibitor; Th, T-helper cell.

**Figure S3.**
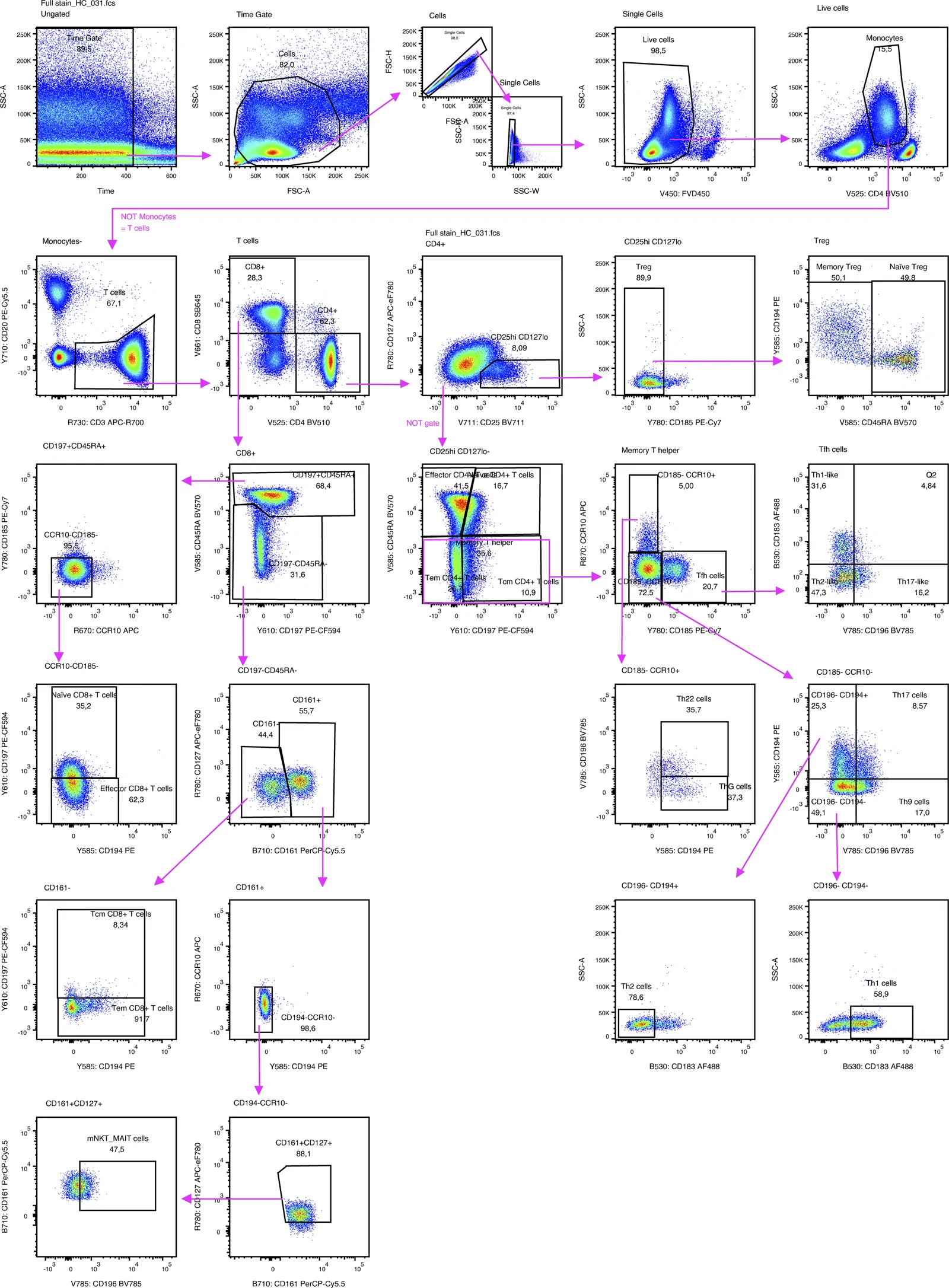
**Flow cytometry gating strategy for T helper cell subset identification.** Multiparameter flow cytometry gating strategy demonstrating sequential identification and characterization of T cell subsets. Software, FlowJo v10. TEM, T effector memory; CCR7, C–C chemokine receptor type 7; CD45RA, cluster of differentiation 45 isoform RA; CD95, cluster of differentiation 95; CD161, cluster of differentiation 161; CD3, cluster of differentiation 3; CD4, cluster of differentiation 4.

To assess the extent of immune reconstitution induced by JAK inhibitors, longitudinal single-cell profiling of peripheral blood mononuclear cells (PBMCs) using Cellular Indexing of Transcriptomes and Epitopes by sequencing (CITE-seq) and flow cytometry was performed at baseline, as well as early and late time points after JAK-inhibitors treatment initiation, complemented by serum cytokine analysis (Fig. 1, C and D). All major PBMC subsets were identified across samples (Fig. S2), revealing baseline predominance of the classical monocyte cluster relative to lymphocytes (Fig. 2, A and B), consistent with conventional laboratory counts and flow cytometry at matched time points (Fig. S1, Fig. S3, and Table S2). Of note, the pronounced leucopenia that had prompted JAK-inhibitor initiation in Patient B 1 wk earlier was still present at the early posttreatment time point (“Early”) (Table S1).

In both patients, B cells and dendritic cells showed variable abundance changes. However, despite treatment, NK cells remained absent in Patient B (Fig. 2 B and Fig. S2). The two patients exhibited the characteristic STAT1 GOF–associated Th17 deficiency at baseline compared with the healthy control (HC). JAK-inhibitor treatment induced a moderate, but incomplete Th17 recovery, from 0.33% to 0.95% of T cells in Patient A and from 2.18% to 3.71% in Patient B, coinciding with rising serum IL-17A levels (Fig. 2, D and E; Table S2; and Fig. S3). The early time point Th17 levels in Patient B also mirrored the yet-unresolved cytopenia. In contrast, gating IL-17–producing cells in the CD8^+^ T cell compartment revealed a persistent absence of mucosal-associated invariant T (MAIT) and mucosal NKT (mNKT) cells, with no reconstitution during treatment (Fig. 2 F). Regulatory T cells (Tregs), both naïve and memory subsets, remained nearly absent (naïve and memory combined <1.5% of total T cells) across all time points in both patients (Table S2 and Fig. S3).

### JAK-inhibitors partially restore naïve and memory T cell subsets

**Figure 3.**
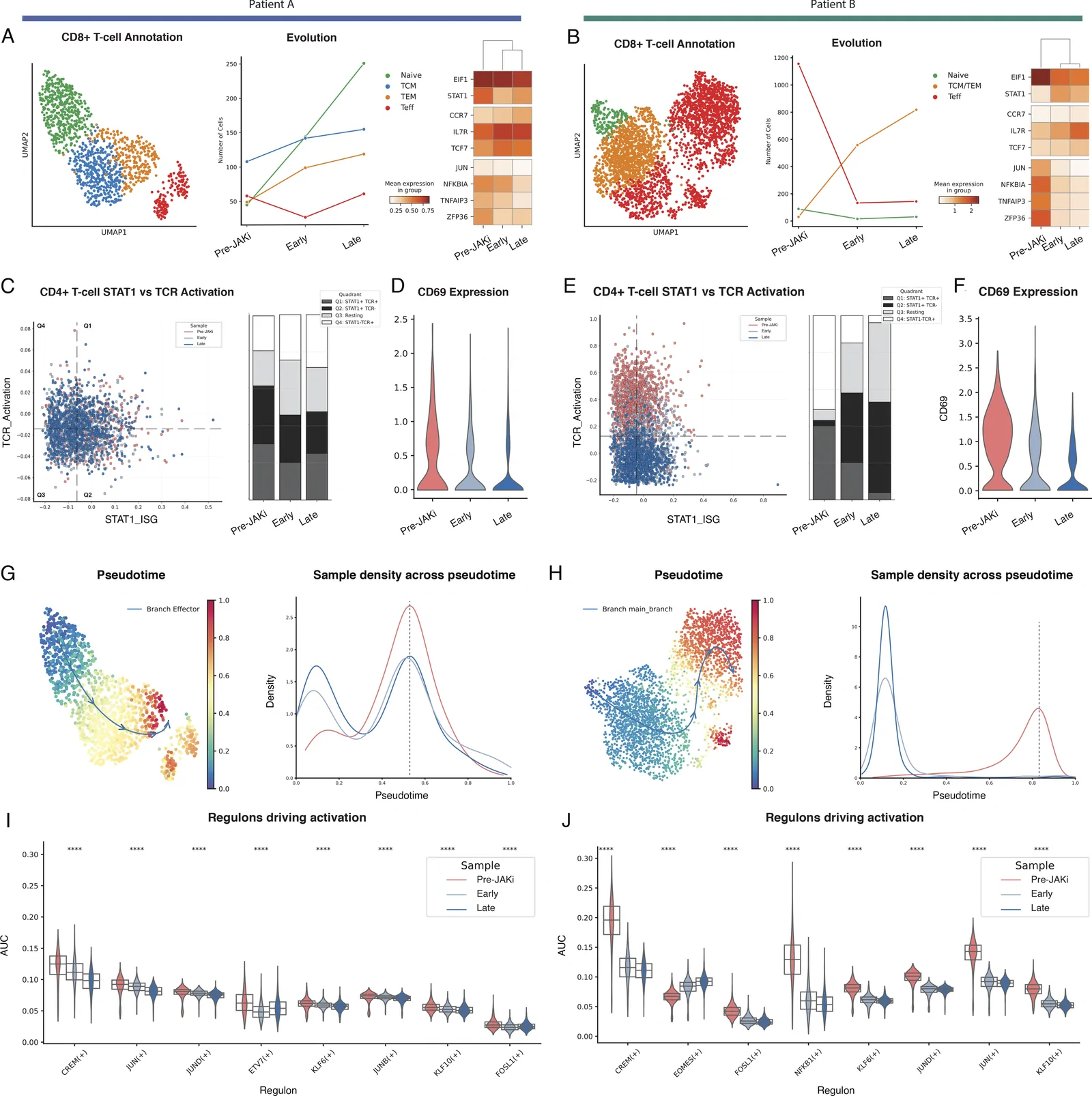
**Annotation, evolution, and analysis of CD4** ^**+**^ **T cell subsets. (A** **–** **C and E)** Depict the annotation and longitudinal evolution of CD4^+^ T cells, with corresponding markers shown in heatmap. Panels C and E show correlation between STAT1 signaling and TCR activation in CD4^+^ T cells. **(D and F–J)***CD69* expression as an early activation marker. Panels G and H show pseudotime trajectories and cell density distributions of CD4^+^ T cells over pseudotime. Panels I and J highlight significant regulons driving treatment progression in CD4^+^ T cell subsets. Pre-JAKi, pre-treatment; early, 1 wk after treatment start; late, 6 mo after treatment start; Teff, effector T cell; TEM, effector memory T cell; CD4, cluster of differentiation 4; TCR, JAK inhibitor, Janus kinase inhibitor; AUC, area under the curve. ****P < 0.0001.

**Figure S4.**
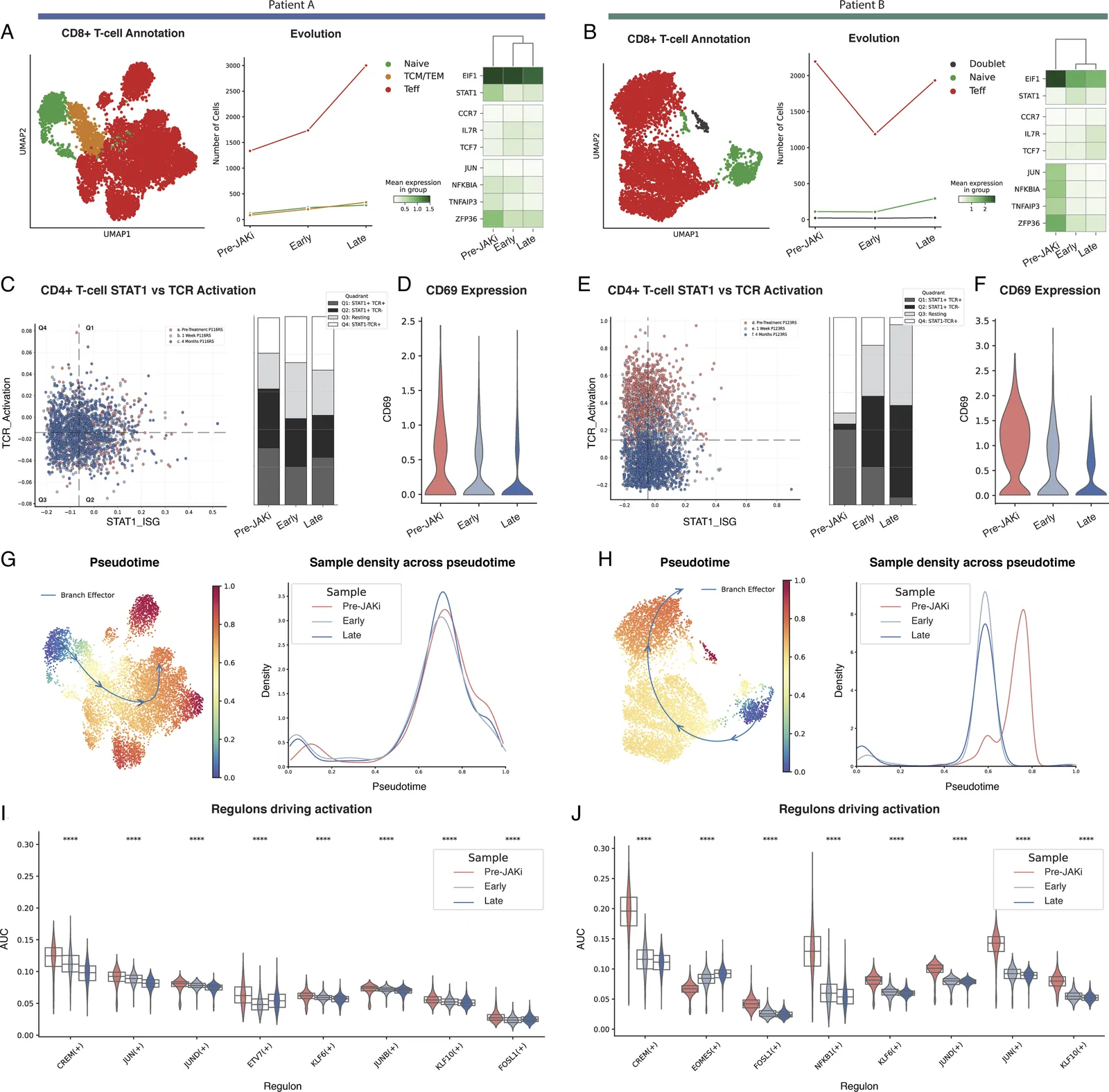
**Annotation, evolution, and analysis of CD8** ^**+**^ **T cell subsets. (A–C and E)** Depict the annotation and longitudinal evolution of CD8^+^ T cells, respectively, with corresponding markers shown in heatmap. Panels C and E show correlation between STAT1 signaling and TCR activation in CD8^+^ T cells. **(D and F–J)***CD69* expression as an early activation marker. Panels G and H show pseudotime trajectories and cell density distributions of CD8^+^ T cells over pseudotime. Panels I and J highlight significant regulons driving treatment progression in CD8^+^ T cell subsets. Pre-JAKi, pre-treatment; early, 1 wk after treatment start; late, 6 mo after treatment start; Teff, effector T cell; TEM, effector memory T cell; CD8, cluster of differentiation 8; JAK inhibitor, Janus kinase inhibitor; AUC, area under the curve. ****P < 0.0001.

Within the T cell compartment, both patients exhibited low CD4^+^ T cells (36–44% of T cells) and expanded CD8^+^ T cells (37–49%) compared with HC (CD4^+^ ∼60%, CD8^+^ ∼28%), resulting in inverted CD4^+^/CD8^+^ ratios that persisted after treatment despite modest increases (Fig. 2C and Table S2). To analyze the effect of JAK-inhibition in T cells, we annotated CD4^+^ (Fig. 3) and CD8^+^ T cells (Fig. S4) by phenotype. At baseline, both patients showed quasi-absence of naïve T cells. In Patient B*,* CD4^+^ and CD8^+^ T cells were strongly skewed toward effector phenotypes (Fig. 3 and Fig. S4). Subsequent clustering, based on gene expression, revealed inflammatory transcriptional signatures at baseline in both lineages, consistent with pre-treatment hyperactivation (Fig. S2). JAK-inhibitor treatment drove modest early increase in naïve (mostly in Patient A) and memory T cells (in both patients). Notably, Patient A had an expanded cytotoxic CD4^+^ T lymphocyte population (*NKG7*^+^*GZMB*^+^) subtype (Fig. S2), suggesting a potential Th1-skewed cytotoxic subset induced in STAT1 GOF.

### JAK inhibitors partially resolve T cell homeostasis

To evaluate the impact of JAK-inhibitor treatment on T cell homeostasis, we assessed the expression of *CCR7*, *IL7R*, and *TCF7*, a set of genes characteristically expressed by naïve T cells/central memory T cells (TCMs) that are documented to play a role in IL-7–dependent survival, transcriptional “stemness,” and lymphoid homing (37). The expression of *CCR7*, *IL7R*, and *TCF7* increased consistently after treatment in both patients (Fig. 3, A and B, heatmap). This shift was accompanied by a reduced expression of baseline-upregulated inflammatory markers, including AP-1 complex (*JUN*) and NF-κB components (*NFKBIA*, *TNFAIP3*) (Fig. 3, A and B, heatmap). *EIF1*, a core eukaryotic translation initiation factor, emerged as the top marker of these inflammatory clusters, suggesting high translational activity. *STAT1* also showed robust upregulation pre-treatment; however, following JAK-inhibition treatment *EIF1* and *STAT1* were downregulated for Patient A, while *STAT1* expression remained dysregulated for Patient B.

Next, to further analyze T cell activation over time, we quantified intrinsic STAT1 activation and TCR stimulation via gene signature scoring as described in Materials and methods, on the scRNA-seq data. At baseline, Patient A’s T cells were evenly distributed across the different STAT1- and/or TCR-activation states. Following treatment, resting T cells, here defined as T cells lacking STAT1- or TCR-activation signatures, increased, while STAT1 activation decreased, and TCR signaling remained partially active (Fig. 3 C). In contrast, Patient B’s baseline T cells exhibited predominant TCR activation with negligible resting T cells, as defined above. JAK-inhibitor treatment largely suppressed TCR activation, expanding resting-like states (Fig. 3 E). In line with this, expression of CD69, an early T cell activation marker upregulated upon TCR engagement (38), decreased in CD4^+^ and CD8^+^ subsets at the early time point after treatment and further at the late time point, while IL2RA (CD25) expression, a late activation marker associated with sustained proliferation, remained undetectable (Fig. 3, D and F).

To continue assessing the dynamics in T cell development at different time points, we performed pseudotime trajectory analysis. Along these trajectories, early pseudotime corresponds to naïve, resting states, whereas late pseudotime represents highly activated, differentiated effector phenotypes. Cells from Patient A were dispersed throughout pseudotime, with increasing density at earlier, resting-like pseudotime after treatment initiation compared with cells before treatment initiation (Fig. 3, G and I). Patient B’s pre-treatment T cells clustered at late pseudotime points reflecting their effector status (Fig. 3 H and Fig. S4), which shifted toward a reduced activity already early after JAK-inhibitor treatment initiation. Reduced expression of CREM, AP-1, and NF-κB transcription factors, along with other factors, was observed to accompany this shift, altogether pointing toward restored homeostasis (Fig. 3 J).

### JAK-inhibition rebalances the TCR repertoire

**Figure 4.**
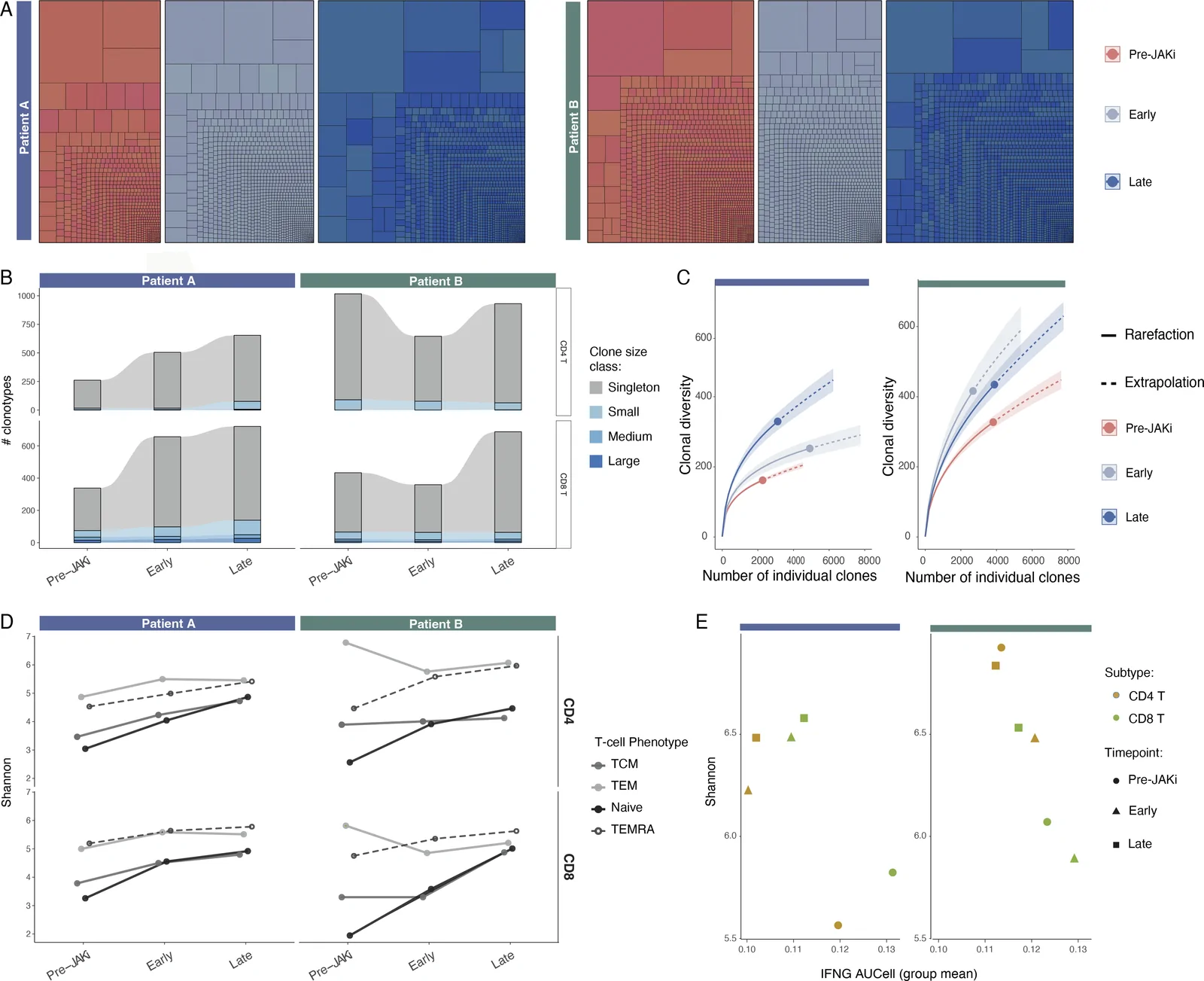
**Evolution of TCR repertoire structure, diversity, and IFN-γ association. (A)** Tree-map representations of TCRβ clonotypes per visit (Pre-JAKi, early, and late) relative to each patient; each rectangle represents a clonotype, rectangle area is proportional to clone size, colored by sampling moment. **(B)** Clone size distribution across time points classified by stacked areas shows the number of clonotypes in each class with overlaid total cell counts, separately for CD4^+^ and CD8^+^ compartments per patient. **(C)** Rarefaction and extrapolation curves estimating clonal richness versus clone number across samples; shading represents 95% confidence intervals; curves allow comparison of expected richness at matched sampling depths. **(D)** Shannon diversity of TCR repertoires by T cell phenotype (naïve, TCM, TEM, and TEMRA) in CD4^+^ and CD8^+^ lineages across time. **(E)** Correlation between Shannon diversity and IFNG AUCell scores across samples, showing per-sample diversity versus inferred IFN-γ pathway activity for CD4^+^and CD8^+^. TEM, T cell effector memory; TEMRA, terminally differentiated effector memory RA^+^; AUCell, area under the curve gene set activity score calculated via AUCell.

**Figure S5.**
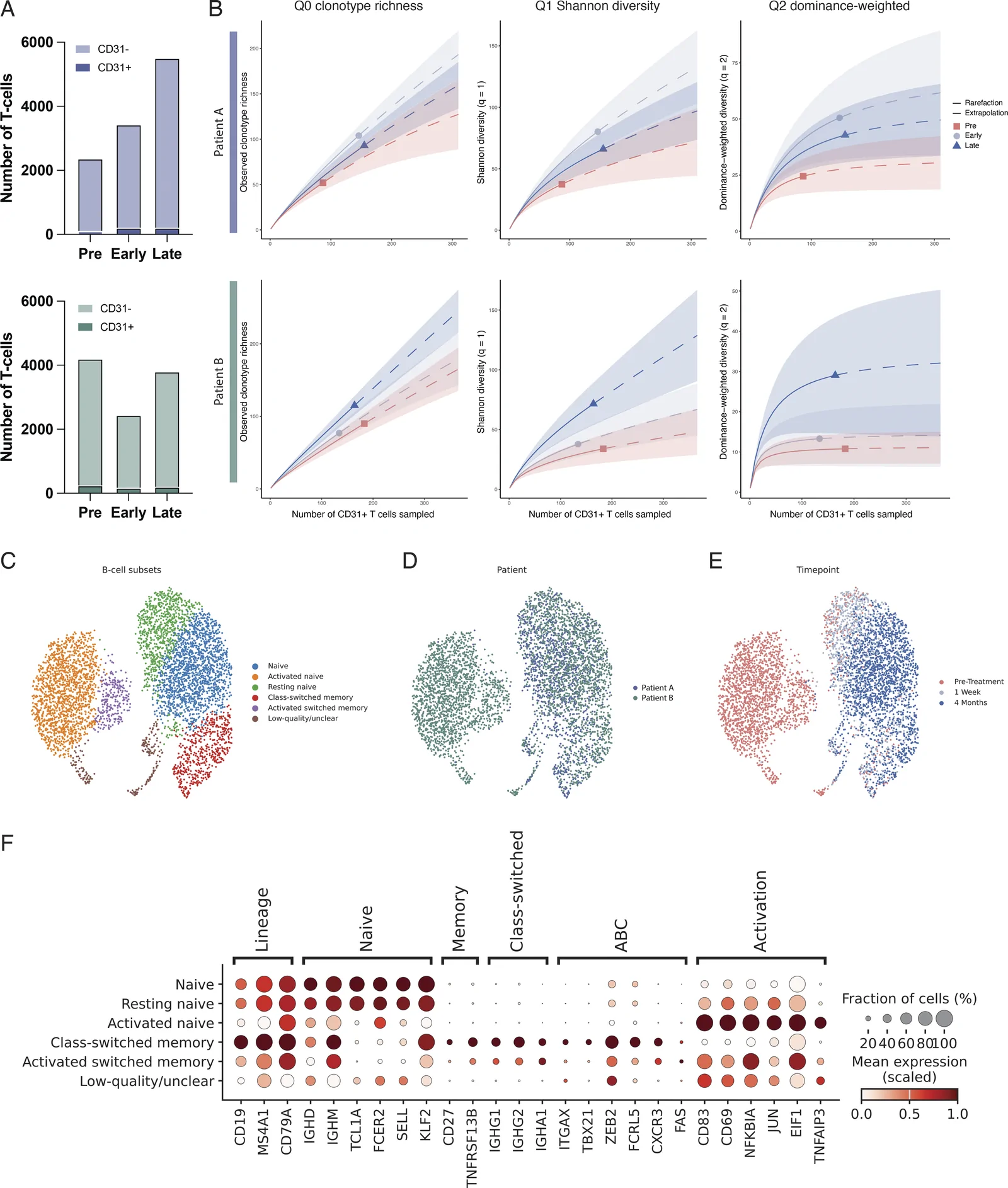
**CD31** ^**+**^ **T cell dynamics and TCR repertoire diversity in recent thymic emigrants and B cell sub-clustering. (A)** Stacked bar plots showing absolute numbers of CD31^+^ (dark) and CD31^−^ (light) T cells, Patient A (top, blue), and Patient B (bottom, green). CD31^+/−^ classification based on surface protein expression from the CITE-seq panel (CD31 > 0.8). **(B)** Rarefaction (solid lines) and extrapolation (dashed lines) curves of TCR repertoire diversity restricted to CD31^+^ T cells, for Patient A (top) and Patient B (bottom). From left to right: Q0 observed clonotype richness, Q1 Shannon diversity, and Q2 dominance-weighted diversity (Hill numbers). Shaded areas represent 95% confidence intervals. Data are shown as individual values (*n* = 1). **(C)** UMAP of peripheral B cells from both patients across all time points, colored by sub-cluster identity. **(D)** UMAP colored by patient. **(E)** UMAP colored by treatment time point. **(F)** Dot plot showing mean scaled expression and fraction of expressing cells for canonical lineage (CD19, MS4A1, and CD79A), naïve (IGHD, IGHM, TCL1A, FCER2, SELL, and KLF2), memory (CD27 and TNFRSF13B), class-switched (IGHG1, IGHG2, and IGHA1), age-associated B cell (ITGAX, TBX21, ZEB2, FCRL5, CXCR3, and FAS), and activation (CD83, CD69, NFKBIA, JUN, EIF1, and TNFAIP3) markers, grouped by B cell sub-cluster.

Because an increase in absolute T cell counts and a decrease in T cell activation suggested quantitative and phenotypic recovery after JAK inhibition, we sought to determine whether this reconstitution reflected the expansion of pre-existing clones or a true restoration of TCR repertoire diversity. To address this, we assessed the TCR repertoire diversity by evaluating clonal expansion and diversity using targeted TCR amplification on paired scRNA-seq. The analysis revealed an overall increase in TCR repertoire diversity following JAK-inhibitor treatment (Fig. 4 A). Clone size distribution analysis showed that the expansion observed in response to JAK inhibitors was dominated by single clones (“singletons”), indicating enhanced repertoire diversity. However, a slight increase was observed in the small clone size class, indicative of clonal expansion, particularly in Patient A (Fig. 4 B). Of note, the pancytopenia observed in Patient B at the early time point following JAK-inhibitor treatment was reflected by lower TCR abundance and diversity in both CD4^+^ and CD8^+^ T cells, with a more pronounced effect on CD4^+^ cells (Fig. 4 B). Rarefaction curves, plotting the number of unique TCR sequences over the number of cells analyzed (Fig. 4 C), showed increased clonal diversity on treatment in both patients. Because the observed richness curves did not reach a plateau, part of the apparent gain reflects the concurrent rise in absolute T cell numbers. To assess whether this diversification involved thymic-derived input, we leveraged CD31 surface expression from the CITE-seq panel to identify putative recent thymic emigrants. The CD31^+^ compartment did not show an expansion but TCR diversity recomputed on CD31^+^ cells at matched sampling depths increased from pre-treatment to the late time point; higher-order Hill metrics, more robust to undersampling, supported the same trend (Fig. S5). These data together with the increase of survival markers in T cells (Fig. 3, A and B, heatmap) indicate that the apparent gain in TCR diversity is driven by an increase in the absolute number of surviving T cells rather than by *de novo* clonal diversification, consistent with enhanced peripheral survival following JAK-inhibitor treatment.

To ascertain the specific T cell subsets that contributed to the repertoire recovery, we calculated the Shannon diversity index within CD4^+^ and CD8^+^ T cell populations (Fig. 4 D). Both patients showed a marked increase in naïve T cell and TCM subsets after JAK-inhibitor treatment, whereas the effector compartment exhibited only a modest expansion. This enhancement in naïve versus effector cell diversity suggests a rebalancing of the TCR repertoire, alongside reduced expansion of the effector T cell population. Interestingly, the IFN-γ transcriptional signature score negatively correlated with the TCR repertoire diversity (Fig. 4 E).

### JAK inhibition attenuates B cell activation and inflammatory programs

**Figure 5.**
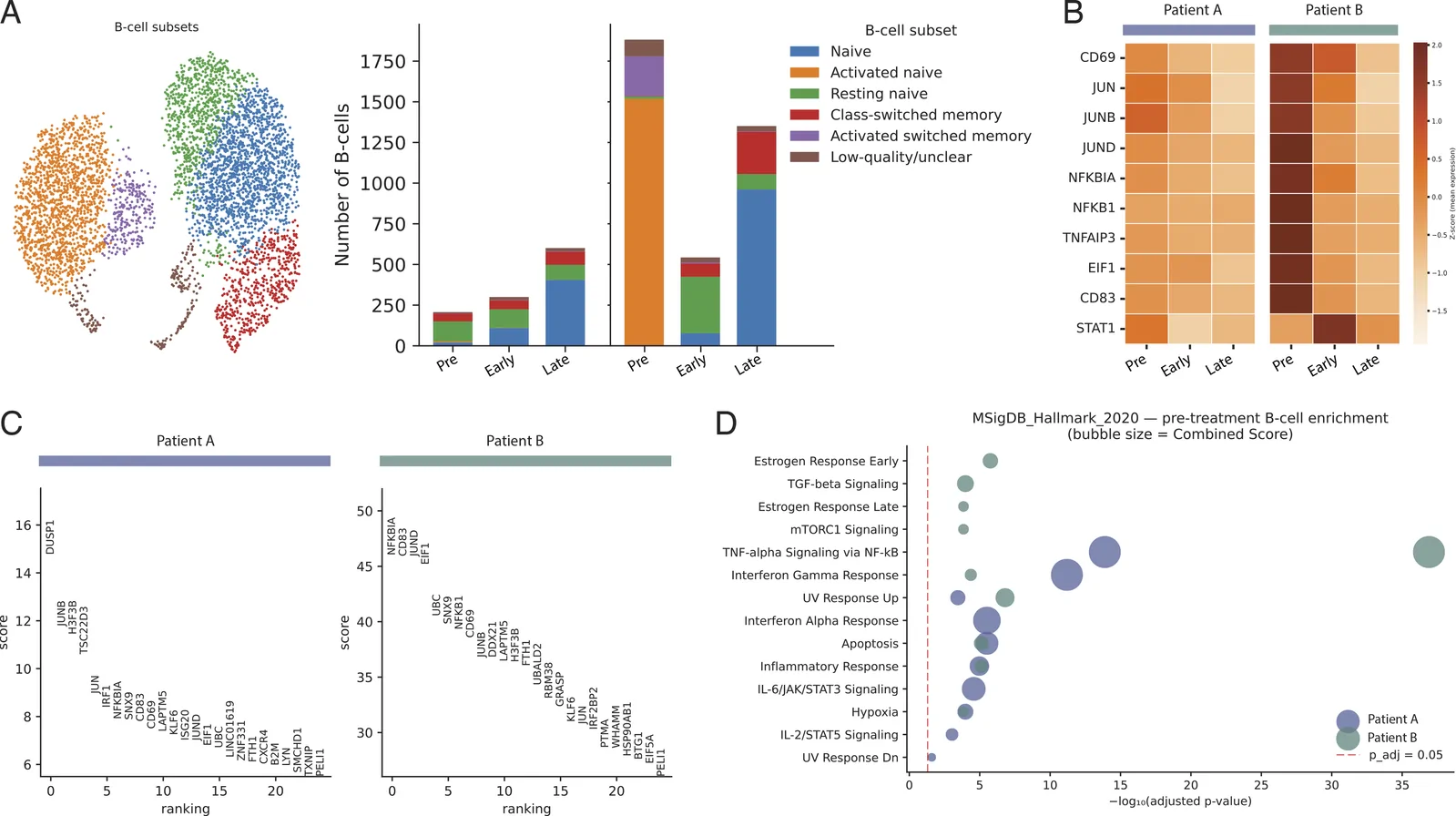
**JAK inhibition reduces B cell activation. (A)** UMAP of peripheral B cells colored by sub-cluster identity (left), and stacked bar plots showing the number of B cells per time point in Patient A and Patient B, colored by B cell sub-cluster. **(B)** Heatmap of mean-scaled expression of activation and inflammatory markers in B cells across time points, per patient. **(C)** Ranked contribution of individual genes to the pre-treatment B cell activation signature in Patient A (left) and Patient B (right); x-axis, gene ranking; y-axis, contribution score. **(D)** MSigDB Hallmark 2020 pathway enrichment of the pre-treatment B cell transcriptome; bubble size reflects combined enrichment score, x-axis shows −log_10_(adjusted P value), dashed red line indicates adjusted P = 0.05. Top-ranked pathways across both patients are shown.

B cell dysregulation has been reported as a marker of more severe disease in STAT1 GOF, and both patients exhibited clinical B cell involvement. Patient A showed specific antibody deficiency and Patient B hypogammaglobulinemia (Table S1). Sub-clustering of CITE-seq B cells resolved six states (Fig. S5). Longitudinal analysis revealed that Patient A had a mixed baseline composition, whereas Patient B showed a predominance of activated naïve B cells that clustered separately and resolved toward a naïve-enriched profile on treatment (Fig. 5 A). Both patients exhibited elevated baseline expression of AP-1 (*JUN*, *JUNB*, and *JUND*), NF-κB (*NFKB1*, *NFKBIA*, and *TNFAIP3*), and activation (*CD69*, *CD83*) programs, together with elevated *STAT1* and *EIF1*, mirroring the T cell activation signature (Fig. 5 B). These signatures decreased on JAK-inhibitor treatment, most markedly in Patient B. Pathway enrichment analysis confirmed significant baseline enrichment of TNF-α/NF-κB, IFN-γ, IFN-α, and IL-6 programs, with the strongest enrichment in Patient B (Fig. 5 C and D). These findings indicate that B cells in STAT1 GOF share the IFN-driven, AP-1/NF-κB–dependent activation signature present in T cells, and that this signature is downregulated by JAK-inhibition. However, longitudinal immunoglobulin values (Fig. S1) and autoantibody screening in Patient B (ANF 1/80, aspecific pANCA without PR3/MPO specificity) indicated that JAK inhibition did not reverse antibody-level defects despite the transcriptional normalization.

### JAK inhibitor suppresses monocyte hyperinflammation

**Figure 6.**
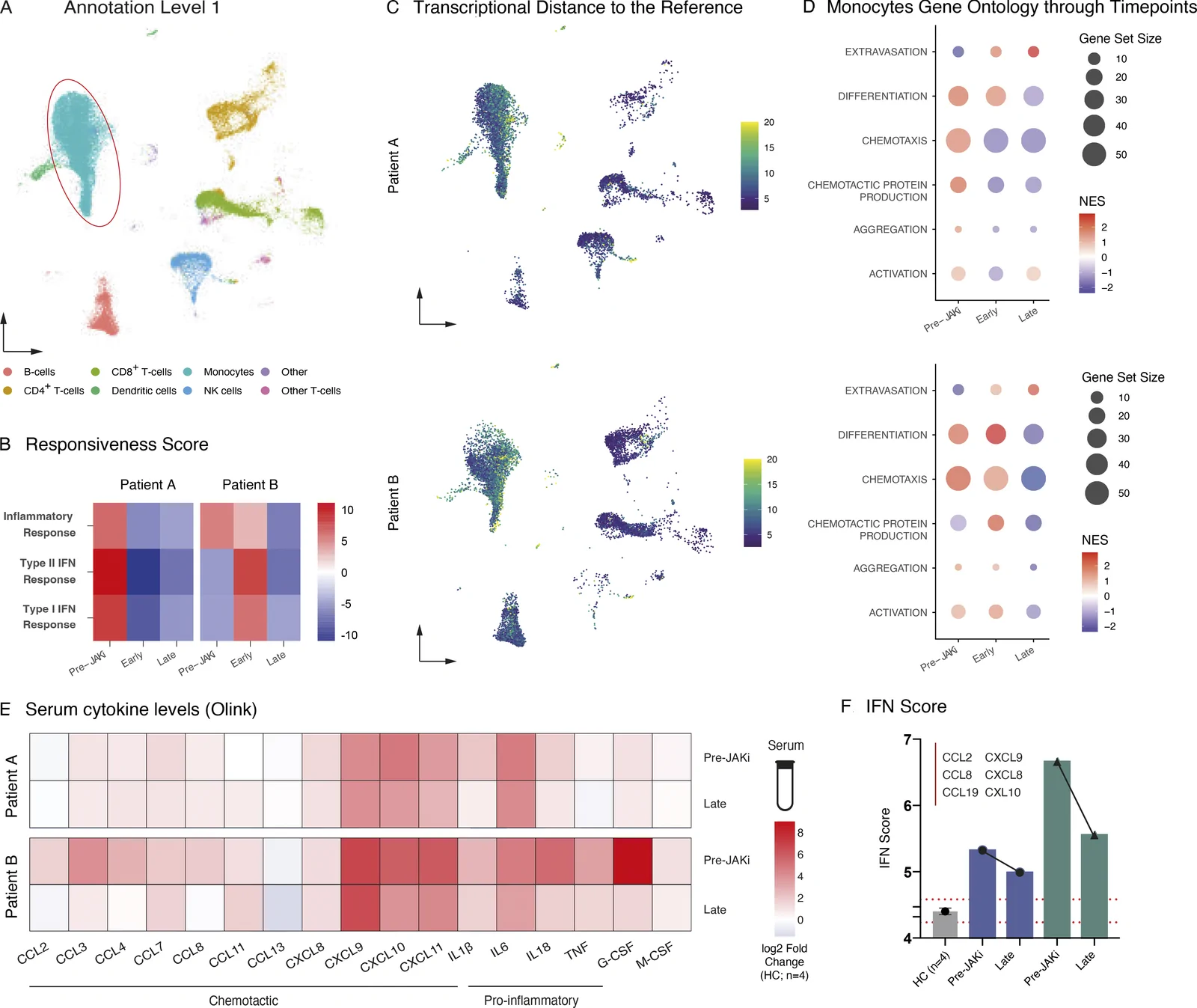
**Monocyte-driven inflammatory activation. (A)** UMAP of PBMC multimodal annotation. **(B)** Module scores of IFN responsiveness and inflammation from MSigDB curated gene sets. **(C)** Transcriptional distance of patient PBMCs compared with a healthy atlas using DISCO database (www.immunesinglecell.com/). **(D)** Gene ontology of monocyte-related gene set from MSigDB. **(E)** Olink serum cytokine levels shown as log2FoldChange compared with HCs (*n* = 4). **(F)** IFN score calculated based on Olink data from CCL2/8/9 and CXCL8/9/10 as described in Di Donato et al. (40).

Next, we examined to what extent monocytes drive inflammation in STAT1 GOF. We mapped transcriptional differences of all cell types to a healthy PBMC atlas (39). At baseline, monocytes from both patients exhibited substantial transcriptional deviation from the healthy atlas, accompanied by elevated inflammation and high type I and II IFN scores, particularly in Patient A (Fig. 6 A–C). Patient B exhibited a delayed reduction in inflammation, which was likely due to his more severe disease condition. Gene set enrichment analysis confirmed sustained enrichment of markers associated with monocyte chemotactic protein production, chemotaxis, and activation at baseline, which sharply declined upon treatment initiation (Fig. 6 D). Consistent with this, serum cytokine profiling revealed elevated baseline levels of IL-1β, IL-6, TNF, IL-18, and the chemokines CXCL10 and CXCL11 (Fig. 6 E). The elevated levels were most pronounced in Patient B, who exhibited the most severe hyperinflammatory phenotype. Finally, systemic IFN activity, scored based on serum cytokine measurements as previously described (40), confirmed elevated baseline IFN scores that were markedly reduced following JAK-inhibitor treatment, although levels did not reach the healthy range. These results were in line with clinical improvements (Fig. 6 F).

### JAK inhibitors attenuate monocyte-driven cross talk with T cells

**Figure 7.**
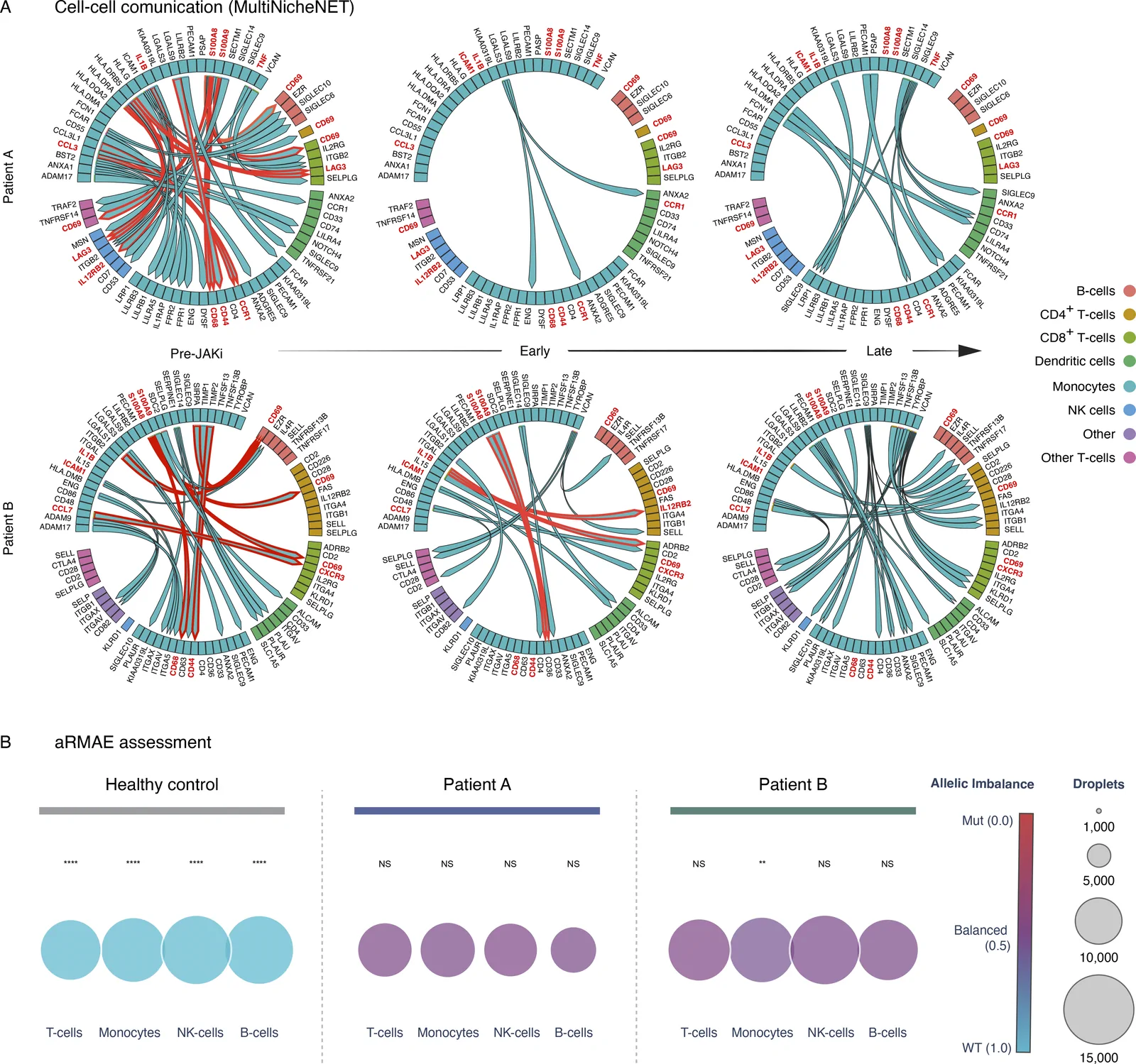
**Cell–cell communication and autosomal random monoallelic expression. (A)** Cell–cell communication analysis of top 50 interactions from monocytes to other immune cells using MultiNicheNet inference pipeline. Interactions shown in red represent activation signals; interactions shown in blue represent other monocyte signaling. **(B)** Bubble plot showing mutant allele fraction in T cells, monocytes, NK cells, and B cells determined by digital droplet PCR. Bubble color represents allelic composition (red represents mutant only, purple represents balanced 50:50, and cyan represents wild-type only), bubble size indicates number of droplets analyzed, and significance markers (****P < 0.0001, **P < 0.01, NS P ≥ 0.01) indicate deviation from biallelic expression (0.50) (two-sided binomial test).

To identify how JAK-inhibitors impact ligand-receptor cross talk between the different T cellular compartments in patients versus HC, we applied MultiNicheNet, an advanced computational framework designed to analyze differential cell–cell communication from multi-sample, multi-condition single-cell transcriptomics data (41, *Preprint*). Before treatment, the top 50 predicted interactions were dominated by pro-inflammatory signaling from monocytes to lymphocyte subsets. These interactions decreased markedly upon JAK-inhibitor therapy. More specifically, S100A8 and S100A9 signaling was reduced, suggesting monocyte-driven inflammation as a central contributor to the hyperactivated T cell state observed at baseline (Fig. 7 A, in red).

### Mutant STAT1 expression remains balanced across the different cell types

A recent study has identified autosomal random monoallelic expression as a potential contributor to incomplete penetrance in IEIs (25), notably in STAT1 GOF. Considering our findings pointing toward a monocyte-driven hyperinflammatory phenotype, we investigated whether cell type–specific expression of the mutant allele in monocytes versus T cells could underlie the observed dysregulated intercellular communication (Fig. 7 B). Patient A showed a balanced allelic expression with fractional abundance of 50.62% in T cells (95% CI: 49.12–52.71%), 49.00% in monocytes (95% CI: 47.24–50.28%), 52.08% in NK cells (95% CI: 50.33–54.11%), and 49.15% in B cells (95% CI: 41.22–56.92%), suggesting biallelic expression across all sorted cell types. Patient B also exhibited biallelic expression in all populations, with mutant fractional abundance of 52.70% in T cells (95% CI: 51.33–53.73%), 44.72% in monocytes (95% CI: 43.40–46.13%), 49.43% in NK cells (95% CI: 47.01–51.66%), and 49.08% in B cells (95% CI: 47.42–50.35%). All measurements remained within the biallelic expression range (20–80%), indicating that both STAT1 alleles are co-expressed in all tested cell types, with no evidence of monoallelic or allele-specific silencing patterns.

## Discussion

This work shows how JAK inhibition leads to a rapid yet incomplete immune reconstitution in two STAT1 GOF patients. Our data reveal that improved T cell homeostasis is accompanied by a rebalancing of the TCR repertoire. Also, monocytes displayed profound dysregulation at baseline, likely contributing to the activated T cell state as hypothesized earlier (5, 35), but this was curtailed by JAK-inhibitor treatment, albeit incomplete and in the absence of autosomal random monoallelic expression.

Patient A harbored a p.R321S mutation in the DNA-binding domain, associated with a classic CMC phenotype (3, 29). Mechanistically, this mutation showed reduced nuclear mobility and slower dephosphorylation (23). Patient B had a p.T385M mutation, linked to a more severe autoimmune phenotype, including increased germinal center formation, enteropathy, cytopenias, and endocrinopathies (42). JAK inhibition successfully resolved CMC in both patients, despite an only modest increase of circulating Th17 and increased IL-17A production (Fig. 1 A; and Fig. 2, D and E). While STAT1 GOF has been associated with suppression of RORγt-dependent Th17 differentiation (43, 44), recent studies on APECED show that exaggerated IFN signaling can impair the epithelial barrier, leading to fungal infection susceptibility (45). However, JAK-inhibitor treatment, acting upstream of the STAT1 GOF mutants, proved insufficient for full reversion of clinical manifestations. Patient A suffered a fatal cardiac event, possibly caused by irreversible tissue fibrosis (36), while Patient B’s autoimmune clinical phenotype showed only partial improvement.

JAK inhibition expanded naïve T cell/TCM subsets, increased TCR repertoire diversity, and dampened IFN and inflammatory programs. However, persistent CD4^+^/CD8^+^ ratio inversion, circulating mucosal-innate T cells (MAIT/mNKT) and Treg depletion, and NK cells recovery failure evidenced incomplete restoration and align with prior evidence of ruxolitinib’s limited effect on MAIT/mNKT and NK maturation correction (32, 46, 47). Restoration of homeostatic marker expression (i.e., *IL7R*, *TCF7*, and *CCR7*) paralleled T cell expansion, supporting the hypothesis that chronic cytokine signaling imposes differentiation pressure on homeostatic pools (48, 49, 50). JAK inhibition appears to relieve this pressure, restoring the IL-7/BCL2-mediated survival and enables a shift toward homeostatic adaptive immune repertoire (51, 52, 53). Both patients gained resting T cells and downregulated CD69, a marker for tissue retention, contributing to chronic tissue inflammation in autoimmune conditions (54, 55, 56). These findings align with JAK-inhibitor effects reported for other conditions where CD69 downregulation relieves aberrant T cell hyperactivity (57, 58, 59).

TCR sequencing revealed that improvement in the T cell compartment resulted from a rebalancing of repertoire diversity rather than expansion of pre-existing dominant clones. Diversity gains were especially evident within naïve T cell and TCM subsets (Fig. 4 D), suggesting that dampening of activation allows expansion of new clones and restoration of homeostatic pools. Because rarefaction curves did not reach a plateau, this apparent gain in diversity is largely accounted for by the concurrent rise in absolute T cell numbers. Analysis of CD31^+^ putative recent thymic emigrants (Fig. S5) indicates that the rebalancing is primarily supported by enhanced peripheral survival rather than *de novo* thymic output. This distinction was limited by the staining efficiency of CD31 detection by CITE-seq. Prior work by Chen et al. reported mono- and oligoclonal repertoires in a cohort of nine patients, using spectratyping of TCR-Vβ (60). Mechanistically, recovery of *IL7R* expression likely supports survival of naïve T cells and TCMs through upregulation of anti-apoptotic BCL2 family proteins, thereby sustaining repertoire diversity (49, 61, 62).

The B cell compartment showed the same AP-1/NF-κB activation signature as T cells and monocytes at baseline (Fig. S5 and Fig. 5), attenuated by JAK-inhibition across all three compartments, supporting a shared cytokine-driven mechanism rather than cell type–autonomous dysregulation. This aligns with Largent and colleagues (13), who identified IFN-γ–driven expansion of CXCR5^lo^CXCR3^+^ B cells correlating with autoantibody titers in STAT1 GOF. Despite this transcriptional normalization, neither patient’s antibody defects resolved during follow-up (Fig. S1), and aspecific autoantibody reactivity persisted in Patient B.

The most pronounced baseline abnormalities originated from the monocyte compartment, which showed elevated type I/II IFN and inflammatory programs. These normalized after JAK inhibition, paralleling serum reductions in IL-1β, IL-6, TNF, IL-18, and CXCL10/11. Ligand-receptor inference (MultiNicheNet [41, *Preprint]*) suggests a model in which myeloid-driven inflammation drives chronic T cell activation (5, 15, 33, 35). Specifically, S100A8/A9 production was highly active, reflecting an alarmin-driven component of inflammation, promoting T cell recruitment and amplification of cytokine signaling. Monocyte inflammation and persistent S100A8/A9 (calprotectin) could serve as biomarkers for residual disease activity as in other auto-inflammatory and autoimmune disorders (63, 64, 65, 66), identifying patients requiring JAK-inhibitor escalation or further immunomodulation.

We explored whether this monocyte-driven phenotype could stem from skewed expression of the mutant allele, as autosomal random monoallelic expression has been described to drive incomplete penetrance in STAT1 GOF and other IEIs (25). However, in our patients, allele-specific expression showed a balanced ratio of mutant/wild-type transcripts across the major PBMC subsets. Biallelic expression demonstrated complete penetrance in these two patients and argued against allelic bias as a driver of the observed dysregulation or a role for skewed expression of the mutated allele in monocyte-driven aberrant intercellular communication, at least in our patients.

### Clinical implications

These data support JAK inhibition as a rapid, pathway-targeted strategy that dampens IFN-driven activation and promotes recovery of T cell repertoire and memory programs, while highlighting measurable residual risks captured by sustained CD4^+^/CD8^+^ ratio inversion, near-absence of Tregs, and mucosal-innate/NK cell deficits. The coordinated reduction of myeloid and lymphoid inflammatory reprogramming together with pro-inflammatory cytokine decline following JAK-inhibitor treatment provides a systems-level rationale for chronic inflammation as the key molecular driver of STAT1 GOF aberrant T cellular dynamics. However, our work also suggests that monocyte hyperinflammation merits further research with potential evaluation of S100A8/A9 or other biomarkers to identify patients with incomplete JAK-inhibitor–mediated control, as in our patients. Thus, although JAK inhibitors are clearly efficacious, they are not a panacea for STAT1 GOF.

### Limitations

The two-patient, PBMC-centric, longitudinal design limits generalization and restricts it to a mere patient description. This likely underestimates tissue reservoirs that sustain antigenic drive. In addition, for Patient A, interpretation of the early versus late JAK-inhibitor response is complicated by an intervening dose increase, making it difficult to disentangle time-dependent effects from dose-dependent effects. While diversity and clonal size metrics improved, causal links between clone-level dynamics, antigen specificity, and clinical outcomes require further exploration. Future studies leveraging longitudinal sampling on more patients with different phenotypes and GOF mutants, and including other affected tissues, or functionally evaluating T cell activation would refine mechanistic understanding of JAK-inhibitor responsiveness. Notably, both patients received relatively low-dose JAK inhibition, and pSTAT1 indices remained variably above the normal range at some time points. This residual fluctuation might indicate incomplete suppression, which could help explain why some symptoms and immunologic abnormalities improved, whereas others persisted.

### Conclusion

By evaluating pro-inflammatory and activated cellular phenotypes, alongside regulatory networks and cell–cell communication, we demonstrate that JAK inhibitors normalize activated phenotypes, increase T cell diversity, and reduce hyperinflammatory signatures, particularly active in monocytes. The absence of allele-specific expression, as previously reported, and residual immune activation suggest that additional factors such as incomplete JAK-inhibitor efficacy, mutation-specific or patient-specific differences influence responsiveness. While JAK-inhibitors are clearly efficacious for many clinical and transcriptional abnormalities, they are not universally corrective for STAT1 GOF. The multimodal framework presented here provides a technical resource for future evaluation of JAK-inhibitor responsiveness.

## Materials and methods

### Sample collection

Patients were diagnosed at the Universitair Ziekenhuis Leuven. After informed consent, consecutive blood and serum samples were obtained during follow-up visits according to a study protocol approved by the UZ Leuven Ethical Committee Review Board (s58466, s69751). Both patients were male adults (55 and 23 years old, respectively). As this was an observational study of two individuals with a rare IEI, randomization, blinding, and formal power calculations were not applicable.

Off-label JAK-inhibitor treatment choice was dependent on availability. Patient A was initiated on baricitinib 2 mg once daily. Due to insufficient therapeutic response, the dosing regimen was adjusted to 2 mg twice daily (BID) during the treatment period. Patient B was treated with ruxolitinib 5 mg BID throughout the study period. Routine assessment of cell counts and pSTAT1 levels was performed. Blood samples were collected prior to the initiation of drug treatment and after 1 wk and 6 mo. Blood was collected by venipuncture using lithium heparin Vacutainers (cat. #366480; BD) and kept at room temperature. Samples were processed within 2 h.

### Blood leukocyte isolation

PBMCs were isolated by density gradient centrifugation using 50 ml Leucosep tubes (Greiner, VWR) according to the manufacturer’s instructions. In brief, blood was diluted 1:2 with sterile PBS solution (D-PBS, Gibco) and placed on top of 15 ml Lymphoprep (STEMCELL) to be then centrifuged for 30 min at 400 *g*. PBMCs and serum phases were collected in 50-ml tubes and washed three times in 50 ml of D-PBS. Cells were manually counted using a Bürker chamber and cryopreserved at 10–15 × 10^6^ cells/ml in cold fetal bovine serum (FBS) with 10% dimethyl sulfoxide (DMSO) in liquid nitrogen.

### Analysis of pSTAT1 by flow cytometry

Peripheral blood cells were stained with anti-CD14 APC and anti-CD45 PerCP (BD) for 30 minutes at 4°C and then incubated either with IFN-γ (15 min, 2,000 IU/ml) or with IFN-α (15 min, 2,000 IU/ml) at 37°C. Next, RBCs were lysed with Lyse/Fix Buffer (558049; BD Phosflow) for 10 min at 37°C and washed with 1 ml of Stain Buffer (554657; BD Pharmingen). Fixed cells were permeabilized with Perm Buffer III (558050; BD Phosflow) for 30 min on ice. After wash, the cells were stained in stain buffer with PE-labeled antibody specific for phosphorylated STAT1 (pY701) (AB_11151907; BD Phosflow) for 30 minutes at 4°C. Phosphorylated STAT1 was evaluated in the CD14^+^ cells gate. Cell acquisition and analysis were performed using a FACSCanto II instrument (BD).

### Analysis of T helper subsets

PBMCs were rapidly thawed in a 37°C water bath and gradually diluted into pre-warmed complete HBSS. Following centrifugation at 300 *g* for 10 min at 20°C, cells were treated with 0.1 mg/ml DNase I for 10 min and then incubated with 1× RBC lysing solution. After washing with PBS, viable and nonviable cells were counted using trypan blue exclusion.

Cells were stained with Fixable Viability Dye eFluor450 (65-0863-18; eBio) at a concentration of 2 × 10^6 cells/ml for 30 min at room temperature in the dark. After washing and blocking with Human BD Fc Block (catalog 564220; BD) at 4°C for 10 min, 1 × 10^6 cells were stained with antibody cocktails or fluorescence-minus-one (FMO) controls for 30 min at 4°C. The antibody panel included CD4 BV510 (562970; BD), CD25 BV711 (563159; BD), CD183 AF488 (558047; BD), CD194 PE (551120; BD), CD197 PE-CF594 (562381; BD), CD185 biotin (552118; BD) with Streptavidin-PE-Cy7 (557598; BD), CD3 APC-R700 (565119; BD), CD8 SuperBright 645 (64-0088-42; Invitrogen), CD20 PE-Cy5.5 (35-0209-42; eBio), CD127 APC-eF780 (47-1278-42; eBio), CD45RA BV570 (304132; BioLegend), CD161 PerCP-Cy5.5 (339908; BioLegend), and CD196 BV785 (353422; BioLegend). Following staining, cells were washed, fixed with 0.5% formaldehyde in PBS for 15 min at 4°C, and incubated with Streptavidin-PE-Cy7 for 30 min at 4°C. Samples were washed again and resuspended in PBS for flow cytometry acquisition.

Flow cytometry was conducted on a BD LSRFortessa instrument in accordance with standard methods. Calibration was performed before each acquisition by CS&T beads (552843; BD). At least 2 × 10^6^ events were acquired for PBMC samples. For fluorescence compensation settings, single-color UltraComp eBeads compensation beads were used (Thermo Fisher Scientific). FMO controls were included.

### scRNA-seq

Libraries were sequenced on a NovaSeq6000 (Illumina) using paired-end reads with 28/10/10/90 cycles for R1/i7/i5/R2 and 1% PhiX spike-in. Sequencing was performed at the VIB Nucleomics Core (VIB). Gene expression reads were aligned to the human reference genome (GRCh38: GENCODE v23/Ensembl98) using CellRanger (10X Genomics – version 7.1.0). Antibody-derived tags were mapped using a custom reference containing the 281 added antibodies. TCR libraries were processed using CellRanger (10X Genomics). Raw sequencing data were demultiplexed and aligned to the human V(D)J reference (10X Genomics – version 7.1.0) using the cellranger vdj pipeline that performs read alignment, assembly of V(D)J sequences, and clonotype assignment for each cell. The resulting output included consensus sequences for TRA and TRB chains, clonotype annotations, and quality metrics.

### Demultiplexing, quality control, and data projection

scRNA-seq analysis was performed in R using the full unfiltered count matrix following the workflow described by the Marioni and Theis laboratories (67). Cells expressing <200 genes and genes detected in <3 cells were excluded.

Hashtag oligo (HTO) data were incorporated using CreateAssayObject (Seurat v4.1.1). HTO counts were normalized using the centered log-ratio method. Demultiplexing was performed using both HTODemux and MultiSeqDemux functions in Seurat. Additionally, samples were demultiplexed using freemuxlet (popscle suite) with default parameters. The input was a filtered matrix derived from the raw matrix. Filtering retained only cells with at least 200 detected genes and genes expressed in a minimum of three cells, same as the Seurat object. Cell assignments from freemuxlet were matched with Seurat demultiplexing results to assign donors based on HTO profiles.

Outlier cells were removed based on library size, gene count, and mitochondrial content. Cells were flagged as outliers when they were five median absolute deviations (MADs) from the median for each metric or 15 MADs for the mitochondrial proportion. These low-quality cells were excluded from the analysis. This filtering strategy was applied to be lenient. The filtered samples were aggregated using the merge function (Seurat v4.1.1), counts were normalized and log2 transformed using the NormalizeData function, both from Seurat using default parameters. Detecting highly variable genes, finding clusters, and creating UMAP plots was done using the Seurat pipeline.

To minimize clustering artifacts arising from highly aberrant transcriptional profiles, all samples were projected onto a subset of KC PBMCs (*n* = 3) profiled by CITE-seq, which served as the reference atlas for label transfer and visualization (68). Cell type annotation was validated by inspecting the expression of canonical lineage-defining surface markers across clusters and samples (Fig. S2). Transcriptional difference to reference was evaluated using the DISCO Webtool for scRNA-seq data integration (39).

### Gene pathway analysis and gene set scoring

STAT1 ISG activity and TCR activation were quantified at the single-cell level using curated gene set module scores computed with scanpy.tl.score_genes, contrasting the mean expression of each signature with a reference gene set per cell. The composition of STAT1/TCR activation states was then assessed by using the global medians of the STAT1-ISG and TCR activation scores as thresholds to assign cells to four quadrants (STAT1+TCR+, STAT1+TCR−, STAT1−TCR−, and STAT1−TCR+) and comparing the proportion of cells in each quadrant. Genes used for calculation of STAT1-ISG and TCR activation can be found in Table S4.

### Trajectory inference analysis

The CD4^+^ and CD8^+^ T cell clusters from each patient were subsetted from the global data set, using the level 1 CITE-seq annotation. The CD4^+^ CTL subset was excluded from downstream analyses to facilitate a more thorough analysis of T helper cell dynamics in response to JAK inhibitors. Each subset was independently normalized, processed for dimensionality reduction, and used for marker gene identification before constructing pseudotime trajectories and cell state evolution over time with palantir (69). To construct the branches, the root T cell was defined according to its expression of naïve-associated genes to end in either a defined population or toward an undetermined endpoint. The resulting pseudotemporal ordering was then used to analyze the regulon activity along pseudotime with pyGAM, identifying significant dynamic regulons (FDR adjusted P < 0.01).

### Serum sampling and Olink cytokine analysis

Blood samples were collected in serum separator tubes (367815; BD) and allowed to clot prior to centrifugation. Samples were centrifuged at 1,200 *g* for 10 min, after which serum was collected and stored at −80°C until further analysis. Prior to cytokine analysis, serum samples were thawed once and processed immediately. Cytokine concentrations were quantified using the Cytokine Target 48 kit from Olink according to the manufacturer’s instructions. Briefly, samples were incubated in the dark at 4°C for 20 h with antibodies coupled to molecular probes. Next, these probes were amplified with PCR using the T100 Thermal Cycler (Bio-Rad), followed by quantification through qPCR in the Olink Signature Q100 instrument. Data quality control was performed using the Olink NPX Signature software (version 1.12.1).

### PBMC sorting and RNA extraction

To assess the effect of STAT1 allelic expression on disease penetrance, pre-treatment PBMCs for the two patients and one healthy donor were thawed in complete HBSS, washed with cHBSS, and stained with CD16 PerCP-Cy5.5, CD3 FITC, CD19 BV421, CD14 APC, and CD56 PE-Cy7 (all BioLegend), with Propidium Iodide (BioLegend) used for viability. For both patients, monocytes, T cells, B cells, and NK cells were sorted into 5-ml tubes containing RLT lysis buffer (Qiagen). RNA from sorted cells was isolated using RNeasy Micro kit (Qiagen).

### Autosomal random monoallelic expression assessment via ddPCR

Custom ddPCR mutation detection assays (Bio-Rad) were designed to differentiate the mutated (FAM) from the wild-type (HEX) alleles for Patient A (c.963A>T, p.R321S) and Patient B (c.1154C>T, p.T385M). Patient and HC cDNA was synthesized using the Reliance Select cDNA Synthesis Kit (Bio-Rad). ddPCR (Bio-Rad) was used to quantify the expression of the mutated and wild-type alleles. Allelic imbalance was determined as previously described in Stewart et al. (25).

### Online supplemental material

The supplementary information file contains four tables and five figures. Fig. S1 shows longitudinal laboratory data of the patients; Fig. S2 shows single-cell annotation; Fig. S3 shows flow cytometry gating strategy; Fig. S4 shows annotation, pseudotime, and regulon analysis of CD8^+^ T cells; Fig. S5 shows TCR diversity of recent thymic emigrants and B cell annotation. Tables S1, S2, S3, and S4 contain extended patient descriptions, flow cytometry values, extended laboratory investigations, and gene sets used for score calculations. Table S1 shows clinical, genetic, and laboratory findings of two patients with STAT1 GOF, treated with a JAK inhibitor. Table S2 shows results of flow cytometry immunophenotyping of T cell subsets. Table S3 shows extended laboratory investigations, including viral serology, autoantibody testing, and hemolysis parameters. Table S4 shows genes used for score calculation.

### Institutional review board statement

Study s69751, “*Ex vivo* evaluation of JAK-inhibitor and gene therapeutical approach in JAK-STAT related disorders - JAKarta study,” ClinicalTrials.gov NCT07261891, drafted by CIH, RS, received approval from the Ethics Committee Research UZ/KU Leuven on 21 November 2024.

### Informed consent

Informed consent was obtained from all patients and donors involved in the study.

## Supplementary Material

Table S1 shows clinical, genetic, and laboratory findings of two patients with STAT1 GOF, treated with a JAK inhibitor.

Table S2 shows results of flow cytometry immunophenotyping of T cell subsets.

Table S3 shows extended laboratory investigations, including viral serology, autoantibody testing, and hemolysis parameters.

Table S4 shows genes used for score calculation.

## Data Availability

The code used in this study can be found at https://github.com/ceciih/jaki_stat1/tree/main. The data underlying this study is not publicly available due to patient privacy constraints related to the small cohort size and the terms of the informed consent. The sequencing processed data objects are available from the corresponding author upon reasonable request at https://doi.org/10.48804/AL7UD3.
