# Supplementary material for: JAK-inhibitor treatment induces rapid yet incomplete immune reconstitution in STAT1 gain-of-function
Source: J Hum Immun. 2026 Oct 9;2(6):e20260009. doi: 10.70962/jhi.20260009 (PMC13651749; doi:10.70962/jhi.20260009)
Supplement: Supplementary file 1 [file jhi_20260009_tables1.docx]

**Supplementary Table S1:**

|  | | **Patient A** | **Patient B** |
| --- | --- | --- | --- |
| **General /Diagnosis** | Sex | Male | Male |
|  | Age (at diagnosis) | 48 years | Early childhood |
|  | Age (at start JAK-inhibitor treatment) | 55 years | 21 years |
|  | Mutated protein domain | DNA binding domain (DBD) | DNA binding domain (DBD) |
|  | p-STAT1 index* | Increased | Increased |
| **Disease Manifestations** | **Infections (prior to JAKi)** | | |
|  | CMC | Oropharyngeal, onychomycosis and esophageal involvement complicated by esophageal stenosis | Esophagitis, stomatitis, anitis, onychomycosis |
|  | Viral | / | *Verruca vulgaris*, chronic EBV viremia |
|  | Fungal (other than CMC) | / | *Pneumocystis jiroveci* pneumonia, possible invasive aspergillosis |
|  | Bacterial | Recurrent respiratory tract infections | Recurrent respiratory tract infections and superficial *S. aureus* infections |
|  | **Immune Dysregulation** | | |
|  | Cytopenia | - | Recurrent episodic pancytopenia including severe neutropenia, worsened by infections |
|  | Enteropathy | - | Unclassified inflammatory bowel disease (IBD-U) with fistulizing anal involvement |
|  | Endocrinopathy | Subclinical hypothyroidism | - |
|  | Dermatitis/eczema | - | Psoriasis inversa |
|  | Lymphoproliferation | - | Splenomegaly |
|  | **Pulmonary disease** | Bronchiectasis | Bronchiectasis |
|  | **Vasculopathy** | No aneurysms on brain MRI angiography | No aneurysms on brain MRI angiography |
|  | **Other** | Idiopathic myocarditis | Solitary kidney, glomerulonephritis, malnutrition, severe growth restriction, osteomalacia/osteoporosis |
| **Immune Phenotyping** | Antibody deficiency | Specific antibody deficiency (SPAD) with normal levels of IgG and subclasses, IgM/IgA/IgE | Hypogammaglobulinemia (decreased IgG and IgA) |
|  | Peripheral blood cells counts and lymphocytic subtyping | Normal, including B-, T- and NK-cells | Recurrent pancytopenia, including severe neutropenia.  Low CD4+, near-absent NK-cells |
|  | Bone Marrow Aspirate/Biopsy | - | Normocellular to hypercellular, severe dyserythropoiesis, absence of neutrophils, increase eosinophil numbers |
|  |  | Negative autoimmune serology | ANF 1/160 (negative subtyping), c-ANCA 1/160 (negative subtyping), positive anti-granulocyte antibodies |
| **Treatment** | **JAK-inhibitor** |  |  |
|  | Type of JAK-inhibitor | Baricitinib | Ruxolitinib |
|  | Starting dosage | 2 mg daily | 10 mg daily |
|  | Maximum dosage | 4 mg daily | 10 mg daily |
|  | Reason to start JAK-inhibitors | Idiopathic myocarditis with congestive heart failure | Immune dysregulation including severe pancytopenia |
|  | **Other chronic therapies** |  |  |
|  | Oral antimycotics | Itraconazole | Nystatin, fluconazole |
|  | Antibiotic Prophylaxis | Azithromycin | Azithromycin, levofloxacin |
|  | Immunoglobulin Replacement | - | Subcutaneous immunoglobulin (SCIg) therapy |
|  | Other | Chronic Heart Failure treatment | IBD-treatment (vedolizumab, mesalazine), G-CSF (before start JAK-inhibitor) |

**Supplementary Table S1: Clinical, genetic and laboratory findings of two patients with STAT1 GOF, treated with a JAK-inhibitor.** Abbreviations: CMC, chronic mucocutaneous candidiasis; EBV, Epstein-Barr virus; JAKi, JAK-inhibitor; IBD-U, unclassified inflammatory bowel disease; MRI, magnetic resonance imaging; SPAD, specific antibody deficiency; IgG, immunoglobulin G; IgM, immunoglobulin M; IgA, immunoglobulin A; IgE, immunoglobulin E; NK-cells, natural killer cells; CD4+, cluster of differentiation 4+ T cells; ANF, antinuclear factor; c-ANCA, cytoplasmic anti-neutrophil cytoplasmic antibodies; p-STAT1, phosphorylated signal transducer and activator of transcription 1; DBD, DNA binding domain; SCIg, subcutaneous immunoglobulin; G-CSF, granulocyte-colony stimulating factor; “-”, not present or not applicable; “/”, not reported or not applicable,* In vitro.
