# Supplementary material for: JAK-inhibitor treatment induces rapid yet incomplete immune reconstitution in STAT1 gain-of-function
Source: J Hum Immun. 2026 Oct 9;2(6):e20260009. doi: 10.70962/jhi.20260009 (PMC13651749; doi:10.70962/jhi.20260009)
Supplement: Supplementary file 2 [file jhi_20260009_tables2.docx]

**Supplementary Table S2:**

| **PatientID** | **HC** | **Patient A** | | | **Patient B** | | |
| --- | --- | --- | --- | --- | --- | --- | --- |
| **Timepoint** | **HC** | **Pre-JAKi** | **Early** | **Late** | **Pre-JAKi** | **Early** | **Late** |
| **T cells count** | 140885 | 146411 | 371043 | 318646 | 252000 | 404000 | 244989 |
| **T cells % of Live cells** | 56.7 | 48 | 64.9 | 52 | 41.4 | 41.8 | 54.7 |
| **CD4+ count** | 87917 | 53502 | 152359 | 118562 | 102255 | 181293 | 97920 |
| **CD4+ % of T-cells** | 62.4 | 36.5 | 41.1 | 37.2 | 40.6 | 44.8 | 40 |
| **Naïve Treg % of T-cells** | 2.26 | 0.27 | 0.21 | 0.15 | 0.83 | 0.11 | 0.1 |
| **Memory Treg % of T-cells** | 2.28 | 1.08 | 0.93 | 0.92 | 0.9 | 1.64 | 1.35 |
| **Naïve CD4+ % of T-cells** | 9.57 | 4.99 | 4.79 | 3.33 | 0.55 | 0.94 | 0.78 |
| **Effector CD4+ % of T-cells** | 23.8 | 1 | 0.78 | 0.79 | 1.75 | 2.76 | 2.99 |
| **TEM CD4+ % of T-cells** | 15.3 | 11.1 | 12 | 12.8 | 31.8 | 35.4 | 30.2 |
| **TCM CD4+ % of T-cells** | 6.22 | 17.5 | 21.8 | 18.9 | 5.59 | 3.9 | 4.46 |
| **Th1-like Tfh % of T-cells** | 1.33 | 0.97 | 2.9 | 1.3 | 1.89 | 1.9 | 2.16 |
| **Th2-like Tfh % of T-cells** | 1.99 | 1.04 | 1.39 | 1.15 | 0.51 | 0.63 | 0.45 |
| **Th17-like Tfh % of T-cells** | 16.2 | 3.72 | 4.32 | 4.79 | 1.63 | 0.45 | 1.03 |
| **Th1 % of T-cells** | 4.28 | 3.83 | 8.82 | 4.91 | 8.04 | 6.19 | 6.33 |
| **Th2 % of T-cells** | 2.94 | 1.97 | 1.52 | 2.13 | 4.02 | 5.79 | 3.9 |
| **Th9 % of T-cells** | 2.52 | 0.29 | 1.01 | 0.68 | 2.89 | 0.61 | 2.16 |
| **Th17 % of T-cells** | 1.27 | 0.16 | 0.33 | 0.41 | 1.15 | 0.39 | 1.29 |
| **Th22 % of T-cells** | 0.36 | 0.0041 | 0.16 | 0.23 | 0.21 | 0.00421 | 0.16 |
| **ThG % of T-cells** | 0.38 | 0.1 | 0.13 | 0.14 | 0.13 | 0.85 | 0.11 |
| **CD8+ count** | 39859 | 71566 | 175999 | 153505 | 103143 | 115142 | 90830 |
| **CD8+ % of T-cells** | 28.3 | 48.9 | 47.4 | 48.2 | 40.9 | 28.5 | 37.1 |
| **Naïve CD8+ % of T-cells** | 6.5 | 9.68 | 9.99 | 9.54 | 4.88 | 3.52 | 5.49 |
| **Effector CD8+ % of T-cells** | 11.5 | 10.8 | 8.5 | 9.91 | 4.36 | 4.43 | 5.63 |
| **mNKT_MAIT % of T-cells** | 2.06 | 0.42 | 0.55 | 0.79 | 0.32 | 0.00668 | 0.03 |
| **TEM CD8+ % of T-cells** | 3.64 | 24 | 23.6 | 23.2 | 30.7 | 20 | 24.9 |
| **TCM CD8+ T-cells** | 0.33 | 3.24 | 4.14 | 4.71 | 0.4 | 0.19 | 0.51 |

**Supplementary Table S2: Results flow cytometry immunophenotyping of T-cell subsets.** T-cells count, absolute T-cell number (cells/μL). T-cells % of Live cells, percentage of T-cells among viable lymphocytes. CD4+ count, absolute CD4+ T-cell number (cells/μL). CD4+ % of T-cells, percentage of CD4+ among T-cells. CD8+ count, absolute CD8+ T-cell number (cells/μL). CD8+ % of T-cells, percentage of CD8+ among T-cells. Naïve, Effector, TEM (effector memory), and TCM (central memory) CD4+/CD8+ (% of T-cells), percentages of naïve, effector, effector memory, and central memory subsets among T-cells. Th1, Th2, Th9, Th17, Th22, ThG (% of T-cells), percentages of T-helper subsets among T-cells. Th1-like, Th2-like, and Th17-like Tfh (T-follicular helper cells; % of T-cells), percentages of T-follicular helper subsets among T-cells. Naïve and Memory Treg (regulatory T-cells; % of T-cells), percentages of regulatory T-cell subsets among T-cells. mNKT_MAIT (mucosal-associated invariant T-cells and mucosal natural killer T-cells; % of T-cells), percentage among T-cells.
