# Supplementary material for: JAK-inhibitor treatment induces rapid yet incomplete immune reconstitution in STAT1 gain-of-function
Source: J Hum Immun. 2026 Oct 9;2(6):e20260009. doi: 10.70962/jhi.20260009 (PMC13651749; doi:10.70962/jhi.20260009)
Supplement: Supplementary file 3 [file jhi_20260009_tables3.docx]

**Supplementary Table S3:**

**Patient A (STAT1 GOF c.963C>T, p.R321S; baricitinib)**

| Test | Reference value | –17M | –4M | +22M | +34M |
| --- | --- | --- | --- | --- | --- |
| Viral testing | | | | | |
| EBV PCR | Negative | Weakly positive (<150 IU/mL) | Positive (473 IU/mL) | Positive (311 IU/mL) | Weakly positive (<150 IU/mL) |
| CMV PCR | Negative | Negative |  |  |  |
| HHV6 PCR | Negative | Negative |  |  |  |
| Parvovirus B19 PCR | Negative | Negative |  |  |  |
| Autoantibody testing | | | | | |
|  |  | **–17M** | **+50M** |  |  |
| ANF screening | Negative | Positive | Dubious |  |  |
| ANF titer | Negative | 1 in 80 | 1 in 80 |  |  |
| Anti-DNA (Farr) | <7 IU/mL | <7 IU/mL | <7 IU/mL |  |  |
| Subtyping | Negative | Negative | Negative |  |  |
| ANCA screening | Negative | Positive | Positive |  |  |
| c-ANCA | Negative | 1 in 640 | Weakly positive |  |  |
| p-ANCA | Negative | Negative | Strongly positive |  |  |
| Anti-PR3 EIA | <2 U/mL | 0.7 | <0.6 |  |  |
| Anti-MPO EIA | <3.5 U/mL | 0.7 | <0.6 |  |  |
| Hemolysis parameters | | | | | |
|  |  | **–15M** | **+41M** |  |  |
| DAT (Direct Coombs) | Negative | Strongly positive (3+) | Weakly positive (1+) |  |  |
| Haptoglobin | 0.3–2.00 g/L | 2.08 | <0.10 |  |  |

**Patient B (STAT1 GOF c.1154C>T, p.T385M; ruxolitinib)**

| Test | Reference value | –6M |
| --- | --- | --- |
| Viral testing | | |
| EBV PCR | Negative | Weakly positive (<150 IU/mL) |
| CMV PCR | Negative | Negative |
| HHV6 PCR | Negative | Negative |
| HHV8 PCR | Negative | Negative |
| Parvovirus B19 PCR | Negative | Negative |
| Measles IgG | Negative | Negative |
| Mumps IgG | Negative | Negative |
| Autoantibody testing | | |
|  |  | **–59M** |
| ANF screening | Negative | Negative |
| ANF titer | Negative | Negative |
| Anti-DNA (Farr) | <7 IU/mL | <7 IU/mL |
| Subtyping | Negative | Negative |
| ANCA screening | Negative | Negative |
| TPO antibodies | Negative | Negative |
| AMA | Negative | Negative |
| ASMA | Negative | Negative |
| ACMA | Negative | Negative |
| LKM antibodies | Negative | Negative |
| Rheumatoid factor | <40 IU/mL | <20 IU/mL |
| Anti-CCP | <7 U/mL | 0.7 |

**Supplementary Table S3.** Extended laboratory investigations including viral serology, autoantibody testing, and hemolysis parameters. Timepoints are expressed in months (M) relative to JAK-inhibitor treatment initiation. Timepoints are expressed as months relative to JAK-inhibitor initiation (M– = pre-treatment; M+ = on treatment). Abbreviations: EBV, Epstein–Barr virus; CMV, cytomegalovirus; HHV, human herpesvirus; ANF, antinuclear factor; ANCA, anti-neutrophil cytoplasmic antibody; c-ANCA, cytoplasmic ANCA; p-ANCA, perinuclear ANCA; PR3, proteinase 3; MPO, myeloperoxidase; EIA, enzyme immunoassay; DAT, direct antiglobulin test; TPO, thyroid peroxidase; AMA, antimitochondrial antibodies; ASMA, anti-smooth muscle antibodies; ACMA, anti-cardiac muscle antibodies; LKM, liver kidney microsomal; CCP, cyclic citrullinated peptide.
