# Supplementary material for: JAK-inhibitor treatment induces rapid yet incomplete immune reconstitution in STAT1 gain-of-function
Source: J Hum Immun. 2026 Oct 9;2(6):e20260009. doi: 10.70962/jhi.20260009 (PMC13651749; doi:10.70962/jhi.20260009)
Supplement: Supplementary file 4 [file jhi_20260009_tables4.docx]

**Supplementary Table S4:**

| Score name | Number of genes | Gene list |
| --- | --- | --- |
| STAT1_ISG | 47 | *ISG15, ISG20, IFIT1, IFIT2, IFIT3, IFI6, IFI27, IFI44, IFI44L, IFITM1, IFITM2, IFITM3, MX1, MX2, OAS1, OAS2, OAS3, OASL, RSAD2, BST2, USP18, XAF1, HERC6, CMPK2, IRF7, IRF9, CXCL9, CXCL10, CXCL11, GBP1, GBP2, GBP4, GBP5, CIITA, HLA-DRA, HLA-DMB, TAP1, PSMB9, IDO1, NOS2, STAT1, SOCS1, IRF1, ANKRD22, BATF2, SERPING1, FCGR1A* |
| TCR_activation | 33 | *FOS, FOSB, JUN, JUNB, ATF3, IER2, EGR1, EGR2, EGR3, NR4A1, NR4A2, NR4A3, BATF, IRF4, MYC, DUSP1, DUSP2, DUSP4, DUSP5, ZFP36, ZFP36L2, NFKBID, CD69, IL2, IL2RA, TNF, IFNG, MIR155HG, RGCC, RGS1, PIM1, CD40LG, TNFRSF9* |

**Supplementary Table S4.** Genes used for score calculation. Gene sets used to calculate the STAT1_ISG and TCR_activation scores in Figures 3 and S4. STAT1_ISG score captures mainly STAT1/interferon-associated transcriptional program combining type I (n = 26) with type II (n = 21). TCR-activation (n = 33). TCR_activation score captures TCR-associated activation, including immediate-early response genes and T-cell activation/effector genes. Gene symbols correspond to the GRCh38 (GENCODE) annotation used for alignment.
